# Re-sensitizing Ampicillin and Kanamycin-Resistant *E. coli* and *S. aureus* Using Synergistic Metal Micronutrients-Antibiotic Combinations

**DOI:** 10.3389/fbioe.2020.00612

**Published:** 2020-06-24

**Authors:** Javier Alberto Garza-Cervantes, Jesus F. Meza-Bustillos, Haziel Resendiz-Hernández, Ivan A. Suárez-Cantú, Oscar Antonio Ortega-Rivera, Eva Salinas, Carlos Enrique Escárcega-González, Jose Ruben Morones-Ramírez

**Affiliations:** ^1^Facultad de Ciencias Químicas, Universidad Autónoma de Nuevo León, UANL, San Nicolás de los Garza, Mexico; ^2^Centro de Investigación en Biotecnologíay Nanotecnología, Facultad de Ciencias Químicas, Universidad Autónoma de Nuevo León, Apodaca, Mexico; ^3^Departamento de Microbiología, Centro de Ciencias Básicas, Universidad Autónoma de Aguascalientes, Aguascalientes, Mexico

**Keywords:** re-sensitizing, synergy, combinations, transition metal, antibiotic

## Abstract

Due to the recent emergence of multi-drug resistant strains, the development of novel antimicrobial agents has become a critical issue. The use of micronutrient transition metals is a promising approach to overcome this problem since these compounds exhibit significant toxicity at low concentrations in prokaryotic cells. In this work, we demonstrate that at concentrations lower than their minimal inhibitory concentrations and in combination with different antibiotics, it is possible to mitigate the barriers to employ metallic micronutrients as therapeutic agents. Here, we show that when administered as a combinatorial treatment, Cu^2+^, Zn^2+^, Co^2+^, Cd^2+^, and Ni^2+^ increase susceptibility of *Escherichia coli* and *Staphylococcus aureus* to ampicillin and kanamycin. Furthermore, ampicillin-resistant *E. coli* is re-sensitized to ampicillin when the ampicillin is administered in combination with Cu^2+^, Cd^2+^, or Ni^2^. Similarly, Cu^2+^, Zn^2+^, or Cd^2+^ re-sensitize kanamycin-resistant *E. coli* and *S. aureus* to kanamycin when administered in a combinatorial treatment with those transition metals. Here, we demonstrate that for both susceptible and resistant bacteria, transition-metal micronutrients, and antibiotics interact synergistically in combinatorial treatments and exhibit increased effects when compared to the treatment with the antibiotic alone. Moreover, *in vitro* and *in vivo* assays, using a murine topical infection model, showed no toxicological effects of either treatment at the administered concentrations. Lastly, we show that combinatorial treatments can clear a murine topical infection caused by an antibiotic-resistant strain. Altogether, these results suggest that antibiotic-metallic micronutrient combinatorial treatments will play an important role in future developments of antimicrobial agents and treatments against infections caused by both susceptible and resistant strains.

## Introduction

Within the last century, the discovery of antibiotics is one of humanity's main medical achievements (Walsh and Wright, 2005). However, the extensive and indiscriminate use of antibiotics has led to the emergence and spread of resistant pathogenic bacteria (Wolska et al., 2012). Highly resistant Gram-negative bacteria—*Pseudomonas aeruginosa, Acinetobacter, Klebsiella*, and *Escherichia* species—have become very difficult to treat pathogens worldwide (Boucher et al., 2009) and are, therefore, part of the ESKAPE pathogens (Pendleton et al., 2013), which also include some Gram-positive bacteria such as methicillin-resistant *Staphylococcus aureus* (Coates et al., 2002; Smith and Romesberg, 2007; Hegreness et al., 2008). Due to the increased occurrence of resistant pathogens, there has been a boost in the number of studies aimed to develop new antibiotic analogs and identify new antibacterial therapeutics and strategies (Wolska et al., 2012). Revisiting current and previous antibiotics is an attractive strategy to overcome bacterial resistance, and one promising approach is the use of antibiotic sensitizers (Marks et al., 2013).

Transition metal species—and remarkably silver compounds (Morones et al., 2005; Morones Ramírez, 2009; Morones-Ramirez et al., 2013; Morones-Ramirez and Gallegos-López, 2014)—lie among the most studied metallo-pharmaceuticals, compounds that involve metals due to their therapeutic action (Ray et al., 2007; Mjos and Orvig, 2014). Nonetheless, silver is not the only transition metal widely used as an antimicrobial agent; metallic micronutrients such as zinc (Stanić et al., 2010; Xu and Imlay, 2012) and copper (Grass et al., 2011) species are also known to exhibit antimicrobial effects. Some other transition metal ions, such as Cu^2+^, Cd^2+^, Co^2+^, Ni^2+^, and Zn^2+^, tend to associate strongly to soft bases, such as sulfhydryl groups found in proteins, and therefore the chemical characteristics of these compounds have been associated to their main toxicity mechanisms (Valko et al., 2005; Lemire et al., 2013). More interestingly, most of these metallic ions are also considered as essential metals and vital micronutrients in eukaryotic cells—since they play vital roles in cellular processes (Volesky and Holan, 1995; Madigan et al., 2009). However, essential metals—when present in relatively high concentrations—become harmful (Nies, 1999; Finney and O'Halloran, 2003; Harrison et al., 2004; Lemire et al., 2013).

As an alternative to overcome the drawbacks of using transition metal ions as antimicrobial agents combined with the low efficiency of current antibiotics against resistant bacteria, the overall minimum inhibitory concentration (MIC) can be lowered by the formulation of proper combinations of metal ions with antibiotics, resulting in effective therapies under the toxicity threshold (Cottarel and Wierzbowski, 2007; Morones-Ramirez et al., 2013). The possibility of reviving inactive drugs, due to the emergence of bacterial resistance, can be achieved by inhibiting the expression of the diverse antibiotic resistance mechanisms; and a strategy to achieve this revitalization of drugs could be the formulation of micronutrient transition metal ions and antibiotic combinations.

In this work, we hypothesized that an appropriate approach to potentiate antibiotic effects was to design antibiotic/micronutrient transition metal ion combinations, using susceptible/resistant *E. coli* and *S. aureus*, as models, to test the effects of these therapeutic combinations. Our results supported the effectiveness of this approach. When combinatorial assays, composed of transition metal ions (Cu^2+^, Cd^2+^, Co^2+^, Ni^2+^, and Zn^2+^) with a β-lactam antibiotic (ampicillin) or an aminoglycoside antibiotic (kanamycin), are tested against both model bacteria, the combinations exhibit a significant enhancement in their antimicrobial properties when compared to the individual assays. Moreover, the combinations are capable of re-sensitizing resistant bacterial strains since we observed that the minimum inhibitory concentration of both transition metal ions and antibiotics, significantly decreased when used in combinatorial assays. We then provide enough evidence *in vitro* and *in vivo* that the compounds and the combinations do not present toxicity at the concentrations at which the combinatorial assays exhibit the re-sensitizing synergistic effect against resistant *E. coli* and *S. aureus*. Finally, we demonstrate that that combinatorial treatments can clear a murine topical infection caused by an antibiotic-resistant strain and therefore providing evidence that antibiotic-metallic micronutrient combinatorial treatments have a potential to be used against infections caused by both susceptible and resistant strains.

## Materials and Methods

### Microbial Strains and Conditions

The bacterial strains used were *E. coli* ATCC 11229 and *S. aureus* ATCC 6538, grown on Luria-Bertani (LB; Difco) and Tryptic soy broth (TSB; BD, Bioxon) respectively, at 37°C-150 rpm for 16 h to obtain an overnight culture.

### Minimum Inhibitory Concentration

The MICs were developed based on the methodology of Andrews (2001) and NCCLS (Cavaleri et al., 2005), with some modifications, as reported previously (Garza-Cervantes et al., 2017), in 96-well-plates Costar(Corning). Stocks of the salts, NiSO_4_·6H_2_O, 3CdSO_4_·8H_2_O, ZnSO_4_·7H_2_O (Productos Químicos Monterrey S.A. de C.V., México), CoCl_2_·6H_2_O, and CuSO_4_·5H_2_O (Desarrollo de Especialidades Químicas S.A. de C.V, México) were prepared at a final concentration of 100 mM using distilled water as vehicle. The antibiotics stock, ampicillin, and kanamycin (AGScientific, California, USA), were prepared following the vendor recommendations. From these solutions, we added the necessary volume to achieve the concentrations of 16 mM and 1,024 ppm of transition metal ion and antibiotic, respectively, within a final volume of 200 μL. Next, serial dilutions were performed by taking 100 μL from every next well with 100 μL of culture media and discarding the last 100 μL. This way, the tested concentrations were 8 to 0.0156 mM for the transition metal ions and 512 to 0.007 ppm of each antibiotic after adding the bacterial inoculum.

To inoculate each test well of MIC assay, an overnight culture (20 h culture incubated at 37°C-150 rpm) of each strain (*E. coli* ATCC 11229, or *S. aureus* ATCC 6538) was diluted 1:250 in fresh medium and incubated until it reached a critical optical density at 600 nm (OD_600_) of 0.2 ± 0.02; adjusting it with fresh medium if necessary to reach a cell concentration. A concentration range of 10^7^-10^8^ cells/mL was reached, supported by plate-counting observations measured by serial dilution method. From this, a 1:100 dilution was made with fresh medium in a 1.5 mL tube, and then 100 μL of this dilution were added to each test well to achieve a final concentration of 10^5^ cells/mL, and then incubated at 37°C-150 rpm.

After 20 h of incubation at these conditions, we measured the optical densities (ODs) of control and treated inoculums, and the MIC determination was the value at which no significant growth was observed (OD_600_ < 0.05). All tests, and their respective control samples, were performed in replicates of three.

### MIC Determination Through Checkerboard Assays for the Antibiotic/Transition Metal Ion Combinations

To unveil the synergistic effects of antibiotics and transition metal ions, checkerboard assays (Orhan et al., 2005; Pillai et al., 2005) were developed from the MICs of each. The antibiotic-transition metal combinations (ATMCs) corresponding to 0, 1/2, 1/4, and 1/8 of their respective individual MICs were tested in 96-well-polystyrene plates. Antibiotic and transition metal ion dilutions, in the culture medium, were prepared to when the needed volume was added, MICs fractions were reached. MIC fractions of each antibiotic were prepared along the abscissa, and transition metal ions MICs fractions were prepared along the ordinate.

Bacterial cultures were grown for 16 h at 37°C-150 rpm, from which a dilution (1:250) was prepared in fresh culture medium and incubated until it reached a critical OD_600_ of 0.20 ± 0.02, adjusting it with fresh medium if necessary, obtaining a concentration range of 10^7^-10^8^ cells/mL, supported by plate-counting observations measured by serial dilution method. A 1:20 dilution of this culture was adjusted in the culture medium, followed by the addition of 20 μL (1:10) of the inoculum to each test well, resulting in an estimated concentration ~10^5^ cells/mL within a final volume of 200 μL. The 96-well-plates were then incubated at 37°C-150 rpm for 20 h. After incubation, the ODs of control and treated inoculums were measured, and the respective values were recorded. Each ATMC, and its corresponding control samples, were tested in triplicate.

### Transformation of Resistant Bacteria Strains

The transformation of *E. coli* and *S. aureus* was performed to obtain resistant bacterial strains to antibiotics included in this work, following the methodology proposed by Sambrook et al. (1989) with some modifications. Twenty milliliters of culture medium were inoculated with an overnight culture and then incubated at 37°C-150 rpm until it reached an OD_600_ of 0.3–0.4. Then the culture was transferred to a 50 mL tube and centrifuged at 6,000 rpm for 10 min. Later it was washed twice, first with 1 mL and the second with 0.5 mL of ultra-pure H_2_O, and both centrifuged at 14,000 rpm for 1 min. Suspending the final pellet in 50 μL of cold, sterile glycerol 10%, keeping them in water at 4 °C until use. From this, about 1 μg of the plasmid (pGEX4T2, pUC57-Amp, or pUC57-Kan) was added to the cells, mixed and reposed for 1 min in cold water. The mixture was added to a 0.2 cm electroporation cell and electroporated at 2,500 V. 1 mL of culture media was added immediately after and transferred to a 1.5 mL tube. The cultures were incubated at 37°C-300 rpm for 1–2 h. Aliquots of these cultures were transferred to agar plates with their respective antibiotic and incubated at 37°C for 24 h.

MICs of all antibiotics and transition metal ions were tested in the resistant strains obtained, but only the ATMCs that were capable of inhibiting susceptible strains were tested in the resistant ones with the same methodology described above.

### Cytotoxicity Assay in Mammalian Cells

Cytotoxicity assays were performed as reported previously (Garza-Cervantes et al., 2017). HaCaT cells (Rat cardiomyoblast, CRL-1446, ATCC, Manassas, VA, USA), a spontaneously transformed human keratinocyte cell line, were cultured in DMEM medium supplemented (sDMEM) with 1 g/L glucose, 4 mM L-alanyl-glutamine, 10% heat-inactivated fetal bovine serum (FBS; Gibco, NY, US), 50 IU/penicillin and 50 μg/ml streptomycin (Sigma, Israel) at 37°C and 5% CO_2_. The MTT assay was performed in triplicates, to measure mitochondrial activity as a viability marker, as described previously (Mosmann, 1983) with little modifications. To expose the cultures to the individual metal ions and the ATMC treatments, 96-well-microplates were seeded with 4 × 10^4^ cells in 100 μl of sDMEM and left to attach overnight. Next, the old medium was replaced with 200 μl of sDMEM previously formulated with the different concentrations of transition metal ions. Ten percentage DMSO was used as a positive control and medium alone as a negative control. After a 24 h exposure, the medium was discarded, and 100 μl of fresh MTT solution (0.5 mg/ml of MTT in DMEM medium without FBS) was added to the cells, and they were incubated for 4 h at 37°C and 5% CO_2_. The MTT solution was substituted with 100 μl of Isopropanol-0.04 N HCl, to dissolve the formazan crystals and the optical density at 595 nm (OD_595nm_) (655 nm in the reference) was measured in an iMark microplate reader (Bio-Rad, Tokyo, Japan). Viability was calculated as the ratio of the mean between the OD_595nm_ of the treated groups and the OD_595nm_ of the negative control.

### *In vivo* Toxicological Study

#### Animals

Male adult Wistar rats (200–250 g) were used and maintained in stainless steel cages with a 12 h light/dark regime. All the experimental animals were handled ethically.

#### Experimental Design

To demonstrate the safety in the use of the metal ion/antibiotic combination proposed in this study, four groups of male adult Wistar rats (200–250 gr) with five animals per group (*n* = 5), were injured as in the infection mode described previously (Kugelberg et al., 2005). The control group was treated with 100 μL saline solution, while tree tested groups were treated with 0.5 MIC of Zn^2+^, kanamycin, and zinc-kanamycin combination, respectively, of the *S. aureus-Kan* strain. All the groups were treated in the damaged zone every 24 h for 3 days, as it was done in the antibacterial test. During the experiment, the rats were kept with food and water ad libitum and at room temperature (24 ± 1°C). Then, 24 h after the third day of treatment, urine samples were collected in vessels attached to metabolic cages (TECNIPLAST, 3700M020) in which the rats were placed. Afterward, with the rats previously anesthetized, blood samples were also obtained to sacrifice the experimental animals finally.

#### Biochemical Assays

To test the kidney and liver function of the rats exposed to metal ion, antibiotic and combination, the urine volume was measured, and with its respective method, the concentration of creatinine (Fabiny and Ertingshausen, 1971; Rartels and Böhmer, 1971), glucose (Trinder, 1966), and proteins (Gornall et al., 1949) in urine were measured using UV-VIS spectrophotometer (Varian, DMS 80). In the case of blood plasma samples, the concentration of creatinine (Fabiny and Ertingshausen, 1971; Rartels and Böhmer, 1971), albumin (Doumas et al., 1997), alanine aminotransferase (ALT) (Kaplan et al., 2003), and aspartate aminotransferase (AST) (Kaplan et al., 2003) were measured by the UV-VIS spectrophotometer.

Likewise, along with the experiment, the appearance of the skin (wound) of the experimental groups exposed to Zn^2+^, kanamycin, and combination were observed and compared with the control group.

### *In vivo* Antibacterial Test

#### Animals

Male adult Wistar rats (200–250 g) were used and maintained in stainless steel cages with a 12 h light/dark regime. All the experimental animals were handled ethically.

The *in vivo* experiment based on a tape stripping infection model (Kugelberg et al., 2005) was done with some modifications, as reported previously (Escárcega-González et al., 2018) The rats were anesthetized with pentobarbital (Pentosedal, MAVER laboratories, México D.F.) injecting intraperitoneally 2.5 ml/kg of body weight. Once anesthetized, the back of the rats was shaved in an area of 3 cm^2^ with a trimmer (Oster, 274 series A, U.S.A.). Then, the hairless skin of the rats in an area of 2.5 cm^2^ was tape stripped five times in succession using an elastic adhesive bandage (Sedasiva®, BSN MEDICAL, Colombia). Damage on the skin was defined as the reddening and glistening of the skin with the absence of regular bleeding. This procedure was done in all the animals used in the experiment. After that, to produce infection, the bacterial inoculation was done by placing in the wound produced with the tape stripping process, 100 μl of a 10^7^ cells/ml *S. aureus-Kan* culture in log phase. The *S. aureus-Kan* culture was obtained from an overnight culture grown as described previously.

#### Experimental Design

The rats were divided into five groups using three animals per cage per experimental group (*n* = 3): the control group (with the wound on the skin without infection and treated with 100 μl of saline solution every 24 h for 3 days in the damaged zone), the infected group (treated in the damaged zone with 100 μL of *S. aureus-Kan* culture), and the Zn^2+^, kanamycin, and Zn^2+^-kanamycin treated group (treated with 0.5 MIC of metal ion, antibiotic and combination, respectively, every 24 h for 3 days after the infection with *S. aureus-Kan* in the damaged zone). During the experiment, the rats were kept with food and water ad libitum and at room temperature (24 ± 1°C).

The rats were anesthetized and sacrificed 24 h after the third day of treatment, and 300 mg of the wound (about 1 cm^2^) were removed and homogenized (BIOSPEC tissue tearor, 398, U.S.A.) with 3 ml of phosphate buffer (100 mg_wound_/ml_buffer_).

Finally, 10 μL of homogenates (to have a 1:100 dilution), were used to carry out a pour-plate technique to determine the colony-forming units (CFU) of living bacteria to compare these results among the study groups.

### Ethics Statement

All animal research was approved by the ethics committee of the animal research facility at the Universidad Autónoma de Aguascalientes. The Animal Care and Use Committee of the Universidad Autónoma de Aguascalientes, adhering to the Official Mexican Regulations (NOM-062-ZOO-1999), evaluated the animal care and experimentation practices and approved protocol 25698-UANL-UAA-2019 to perform the experiments. Mexican regulations are in strict accordance with the recommendations in the Guide for the Care and Use of Laboratory Animals of the NIH and the Weatherall Report in the USA to ensure compliance with established international regulations and guidelines. At the end of experiments, animals were sacrificed by using an excess of sodium pentobarbital anesthesia (40 mg/kg bw). Efforts were made to minimize suffering.

### Data Analysis

To estimate the significance of differences between assays employed, all collected data were subjected to analysis of variance (ANOVA) and Fisher's least significant difference (LSD) tests, using Microsoft Excel 2013.

The interaction of antibiotics with transition metal ions was analyzed using the Bliss independence model described by Hegreness et al. (2008), which states that the interaction can be considered as synergistic when the combined effect of the antimicrobial agents is greater than the predicted effect of its components. Thereof, the *S*-value, the difference between the predicted value of individual components x and y (fx0 and f0y, respectively) and the combined treatment xy (fxy) is denoted in the form:

(1) $$\begin{array}{r} S=\left(f_{x0}/f_{00}\right)\left(f_{0y}/f_{00}\right)\;-\;f_{xy}/f_{00} \end{array}$$

Where: *f*_*x*0_ = treatment with antibiotic, *f*_0*y*_ = treatment with metal ion, *f*_*xy*_ = treatment antibiotic+metal ion, *f*_00_ = growth control; *S* > 0 *Synergic, S* = 0 *Aditive and S* < 0 *Antagonic*.

## Results

### Antimicrobial Activity of Metal Ions and Antibiotics in Susceptible and Transformed Bacteria

We tested five transition metal ion micronutrients and two antibiotics -ampicillin and kanamycin- at different concentrations to identify the minimum inhibitory concentration of each compound. Table 1 displays the summary of the observed MIC values. The MICs of the micronutrient transition metal ions are within a range of 1–8 mM for both bacterial strains tested in this work. Once we observed and recorded the MICs of the susceptible bacteria strains, we proceeded to transform these strains to give them the ability to grow in the presence of either ampicillin or kanamycin. As shown in Table 2, the MICs of the resistant strains are considerably higher than the MICs of the non-resistant strains.

**Table 1:** Minimum inhibitory concentrations of transition metal ions and antibiotics.

**Antimicrobial**	***E. coli***	***S. aureus***
Zn^2+^	2 mM	8 mM
Cd^2+^	1 mM	4 mM
Ni^2+^	2 mM	4 mM
Cu^2+^	8 mM	8 mM
Co^2+^	1 mM	2 mM
Ampicillin	32 ppm	0.25 ppm
Kanamycin	32 ppm	8 ppm

*MICs for each of the transition metal ions and antibiotics were determined under the same culture conditions*.

**Table 2:** Minimum inhibitory concentrations of transformed bacteria strains.

**Bacteria**	**Plasmid**	**Resistance**	**MIC (ppm)**	**Fold increase**
*E. coli-Amp*	pGEX4T2	Ampicillin	25,000	781
*E. coli-Kan*	pUC57-Kan	Kanamycin	8,200	256
*S. aureus-Amp*	pUC57-Amp	Ampicillin	1	4
*S. aureus-Kan*	pUC57-Kan	Kanamycin	256	32

*MICs for each antibiotic was determined under the same culture conditions as for susceptible trains*.

### Toxicity Data for the Different Transition Metal Ions

**Figure 1:**
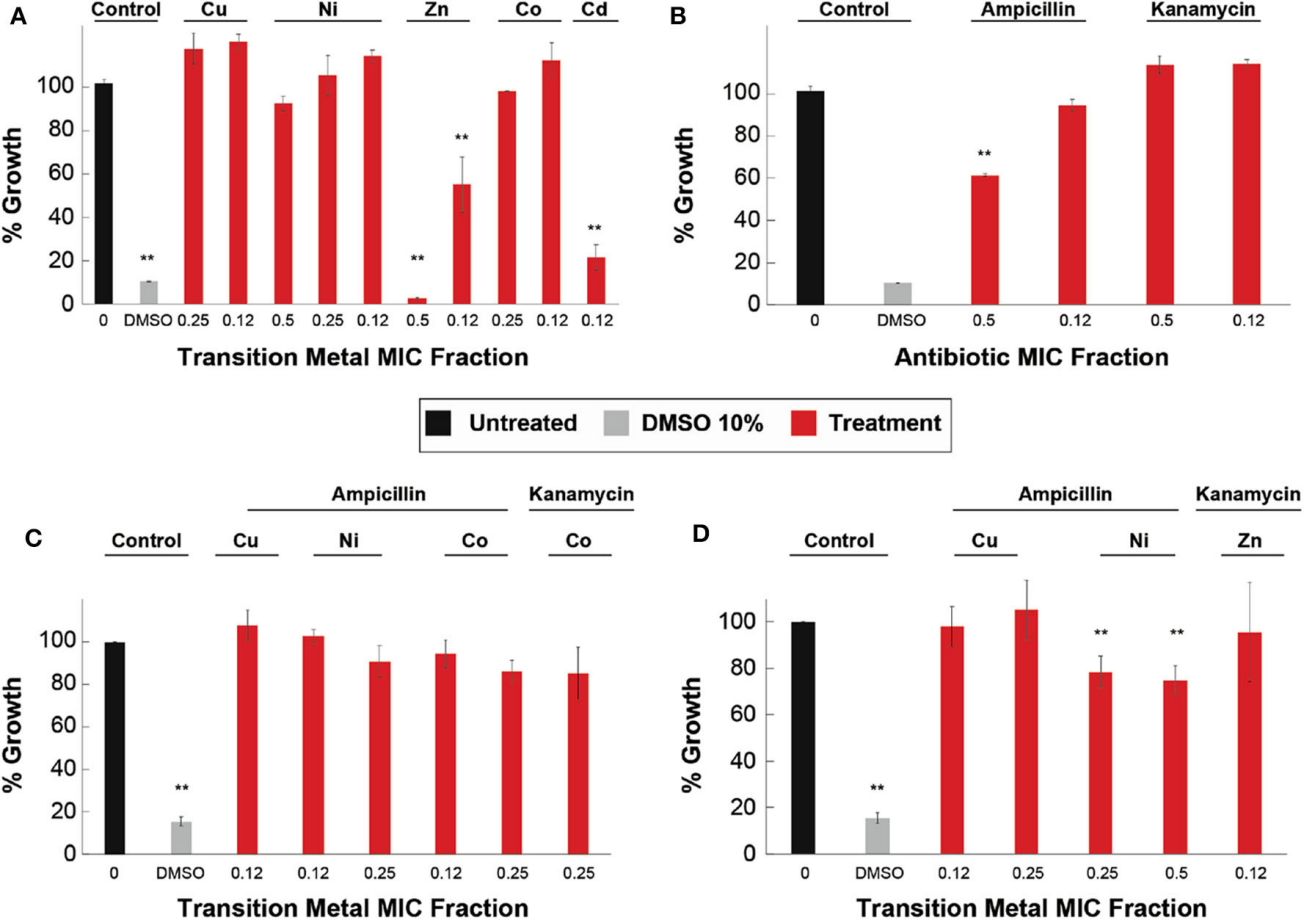
Cytotoxicity of metal ions, antibiotics, and ATMCs on HaCat cells. The effect in cell viability of HaCat cells when exposed to **(A)** transition metal ions, **(B)** antibiotic at resistant strains concentrations, **(C)** ATMCs of susceptible *E. coli* strain, and **(D)** ATMCs of resistant *E. coli* strains. **Corresponds to a significant difference (*p* < 0.05) concerning the control and each of the individual treatments. Error bars correspond to the standard deviation from experiments performed in triplicates.

We tested the toxicity effects of both the antibiotics alone and the antibiotic-transition metal ion combinations, in HaCat cells, at the concentrations used in the antimicrobial assays performed on the resistant strains. The results for the metal ions show that Ni^2+^ was not toxic within the range of the MICs tested in this work (Figure 1A); Cu^2+^ and Co^2+^ exhibited cell viability similar to the control at 0.25 and 0.12 MICs (Figure 1A). For the case of Zn^2+^, there was moderate toxicity at 0.12 of its MIC and high toxicity at 0.5 MIC (Figure 1A). On the other hand, Cd^2+^ showed high toxicity starting at 0.12 MIC (Figure 1A). Moreover, the toxicity assays of the antibiotics, at concentrations corresponding to the MIC fractions of the resistant strains, show that ampicillin has moderate, significant toxicity at 0.5 of its MIC, but exhibits no significant toxicity at 0.12 of its MIC (Figure 1B). Finally, the toxicity assays with kanamycin show no toxicity observed within the concentration range corresponding to the antimicrobial assays performed on the kanamycin resistant strains (Figure 1B).

The results displayed in Figure 1C show that none of the combination treatments (0.12 MIC of Cu^2+^, 0.12, and 0.25 MIC of Ni^2+^ and Co^2+^, in combination with 0.5 MIC of ampicillin, as well as 0.25 MIC of Co^2+^ with 0.5 MIC of kanamycin) exhibited significant differences from the untreated HaCat cells (control), suggesting there is no cytotoxicity linked to these combinatorial treatments. Furthermore, we observed no toxicity in any of the combinatorial treatments (0.12 and 0.25 MICs of Cu^2+^ with 0.5 MIC of ampicillin) tested at concentrations corresponding to those used in the antimicrobial assays against resistant strains (Figure 1D). Moreover, both Ni^2+^-ampicillin combinations showed low toxicity, although significantly different from the untreated cells, with >70% cell viability. Finally, kanamycin combinations composed of 0.12 MIC of Zn^2+^ and 0.5 MIC of kanamycin, showed no significant difference from the untreated cells (control) suggesting no cytotoxicity of the combination.

### Antimicrobial Effect of Antibiotic/Micronutrient Transition Metal Ion Combinations

Once we determined the MICs of each antibiotic and transition metal ion, we proceeded to test the ATMCs through a checkerboard methodology. We tested five transition metal ions in combination with two different antibiotics, to give a total of nine combinations for each metal ion-antibiotic treatment, against two antibiotic-sensitive bacteria. The treatments for *E. coli* showed that four transition metal ions enhanced the antimicrobial activity of each antibiotic. Cu^2+^, Co^2+^, Ni^2+^, and Cd^2+^ potentiated ampicillin and Cu^2+^, Co^2+^, Cd^2+^, and Zn^2+^ potentiated kanamycin. For the treatments with *S. aureus*, only two transition metal ions, Ni^2+^ and Co^2+^, enhanced the antimicrobial activity of ampicillin, however, all of the metal ions tested in this work enhanced the activity of kanamycin. A summary of these results is displayed in Table 3.

**Table 3:** Total of significant interactions between transition metal ions and antibiotics.

**Transition metal ion**	***E. coli***	***S. aureus***	***E. coli***	***S. aureus***	***E. coli-Amp***	***E. coli-Kan***	***S. aureus-Amp***	***S. aureus-Kan***
	**Amp**		**Kan**		**Amp**	**Kan**	**Amp**	**Kan**
Cu^2+^	3	0	2	4	3	1	-	4
Zn^2+^	0	0	2	1	-	5	-	9
Co^2+^	3	2	2	1	0	0	-	0
Cd^2+^	3	0	5	6	7	9	-	6
Ni^2+^	2	3	0	1	2	-	0	-

*Amp, Ampicillin; Kan, Kanamycin; -, Not tested*.

**Figure 2:**
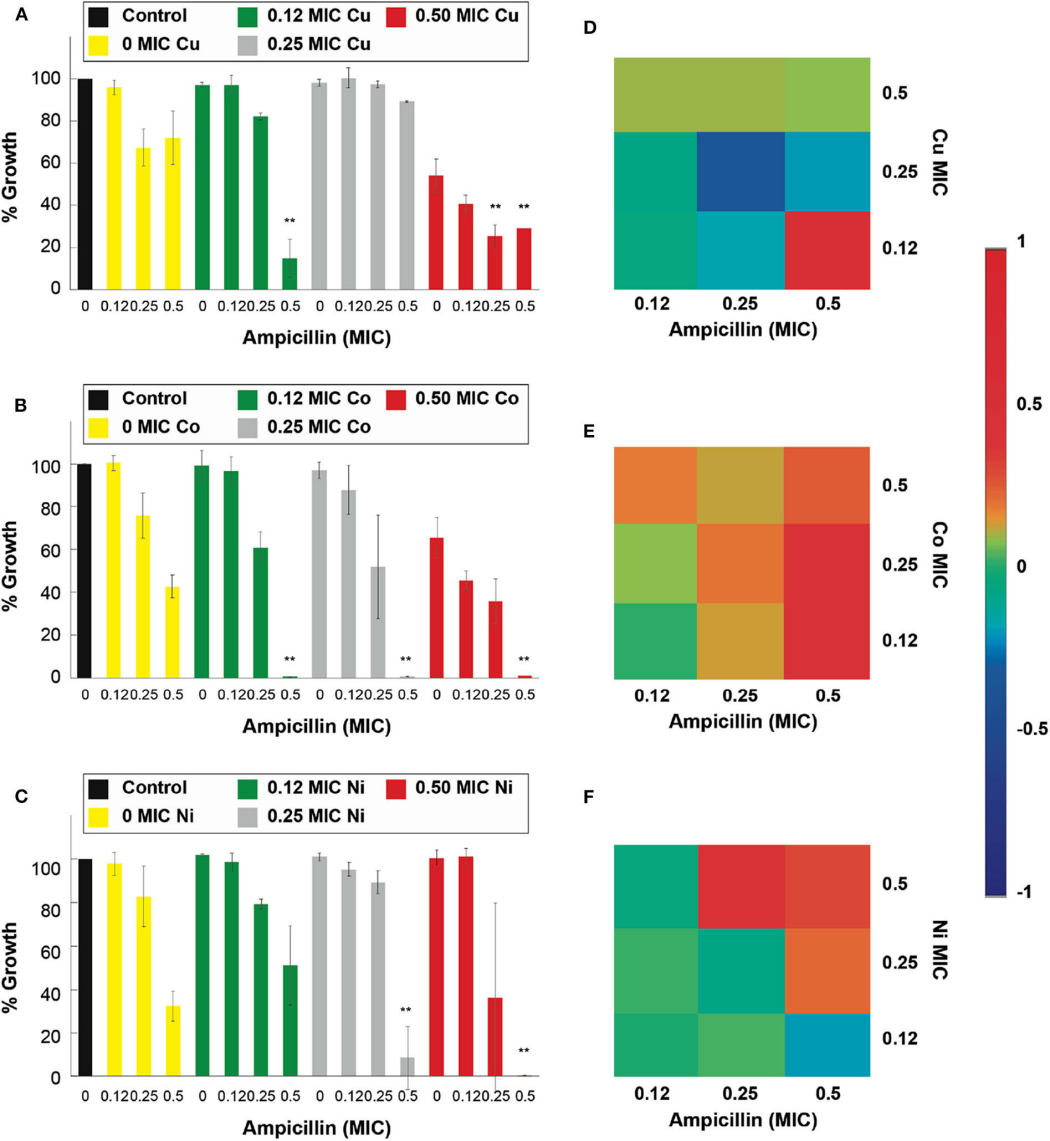
Antimicrobial effect and interactions of ATMCs in *E. coli*. Growth percentage by sub-inhibitory concentrations of metal ion-antibiotic combinations: **(A)** Cu^2+^-ampicillin, **(B)** Co^2+^-ampicillin, and **(C)** Ni^2+^-ampicillin against *E. coli* ATCC 11229. Classification of the different interactions between metal ion-antibiotic combinations. The interactions of **(D)** Cu^2+^-ampicillin, **(E)** Co^2+^-ampicillin, and **(F)** Ni^2+^-ampicillin combinations are classified as synergistic, additive or antagonist, value >0, =0, and <0, respectively. Each experiment was done in triplicates. **Corresponds to a significant difference (*p* < 0.05) concerning the control and each of the individual treatments. Error bars correspond to the standard deviation from experiments performed in triplicates.

In detail, Figure 2 shows that ampicillin, when combined with Cu^2+^, Co^2+^, Ni^2+^, and Cd^2+^, can have a significant (*p* < 0.05) inhibitory effect against *E. coli* when compared with the respective controls (antibiotic and micronutrient transition metal ion alone). At 0.5 MIC of ampicillin, these four metal ions enhanced the effect of the β-lactam antibiotic, mostly at 0.5 and 0.25 of their MICs. Cu^2+^ enhanced significantly (*p* < 0.05) the antimicrobial effect of the antibiotic at 0.5 and 0.12 of its MIC (Figure 2A). Co^2+^ improved the effect of ampicillin at 0.5, 0.25, and 0.12 of its MIC (Figure 2B). Ni^2+^ potentiated the effect of ampicillin at 0.5 and 0.25 of its MIC (Figure 2C). Lastly, Cd^2+^ potentiated the effect of ampicillin at 0.5, 0.25, and 0.12 of its MIC (SFigure 1A). However, Zn^2+^ was not capable of potentiating the antimicrobial effect of ampicillin at the concentrations range tested in this work (SFigure 1B).

**Figure 3:**
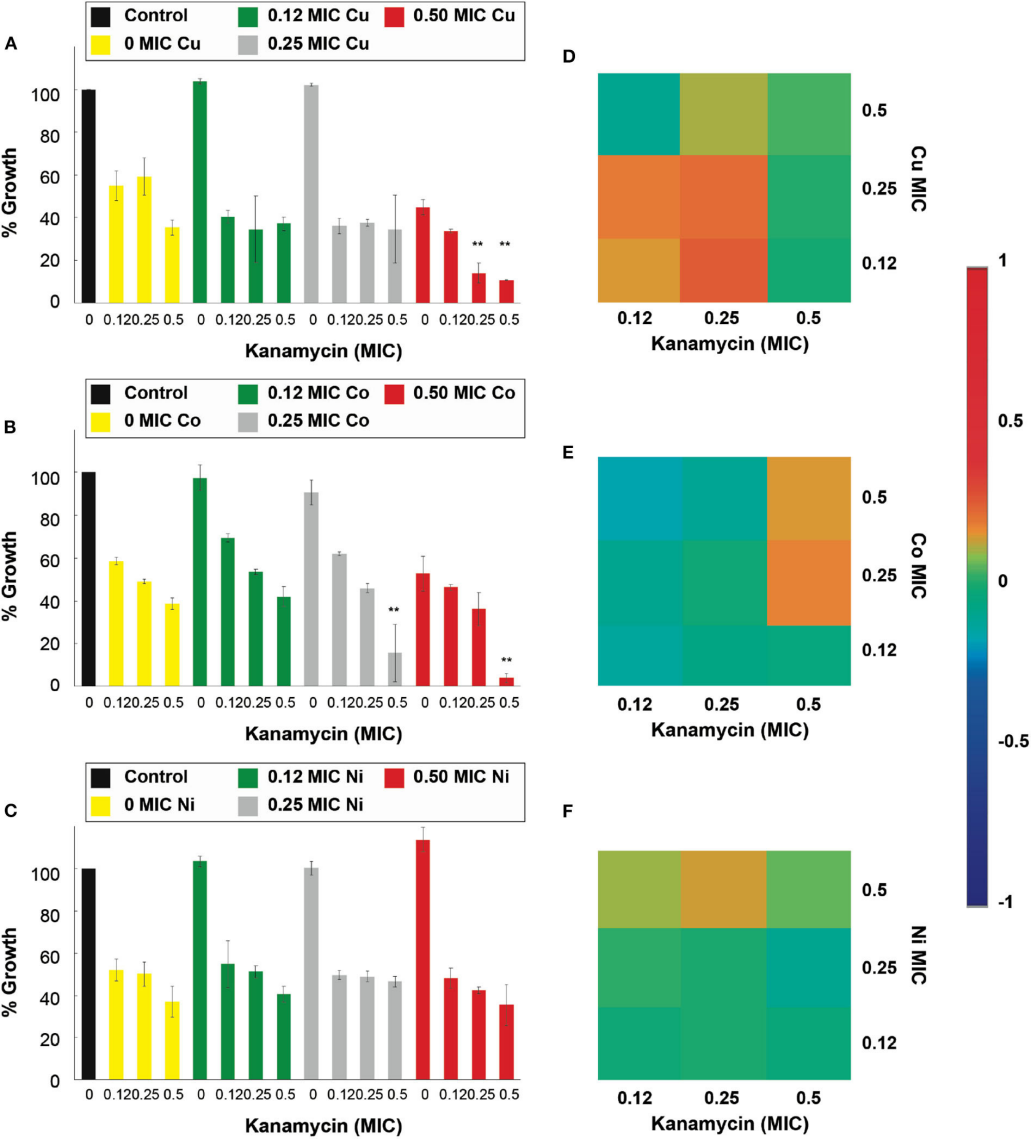
Antimicrobial effect and interactions of ATMCs in *E. coli*. Growth percentage by sub-inhibitory concentrations of metal ion-antibiotic combinations: **(A)** Cu^2+^-kanamycin, **(B)** Co^2+^-kanamycin, and **(C)** Ni^2+^kanamycin against *E. coli* ATCC 11229. Classification of the different interactions between metal ion-antibiotic combinations. The interactions of **(D)** Cu^2+^-kanamycin, **(E)** Co^2+^-kanamycin, and **(F)** Ni^2+^-kanamycin combinations are classified as synergistic, additive or antagonist, value >0, =0, and <0, respectively. Each experiment was done in triplicates. **Corresponds to a significant difference (*p* < 0.05) concerning the control and each of the individual treatments. Error bars correspond to the standard deviation from experiments performed in triplicates.

The results in Figure 3 show that the antimicrobial effects of kanamycin are potentiated significantly (*p* < 0.05), in treatments with *E. coli*, when combined with Cu^2+^, Co^2+^, Cd^2+^, and Zn^2+^. More specifically, a combination of 0.5 Cu^2+^ MIC with 0.5 and 0.25 MIC kanamycin shows enhanced antimicrobial effects (Figure 3A), like the results observed for a 0.5 and 0.25 Co^2+^ MIC combined with a 0.5 kanamycin MIC (Figure 3B). For the case of kanamycin, Ni^2+^ was not capable of potentiating its antimicrobial effect at the range of concentrations tested in this work (Figure 3C). All the MIC fractions for Cd^2+^ enhanced the effect of 0.5 kanamycin MIC, and the 0.5 and 0.25 Cd^2+^ MIC also potentiated the 0.25 kanamycin MIC (SFigure 1C). The effects observed for the combinations with Zn^2+^ resemble the effects with Co^2+^, where 0.5 and 0.25 of its MIC potentiate at 0.5 kanamycin MIC (SFigure 1D).

The nature of the potentiation effects between ampicillin and kanamycin, with micronutrient transition metal ions, are mostly synergistic for the combinatorial antimicrobial treatments tested in the antibiotic-sensitive *E. coli*. Figures 2D–F show that, according to the Bliss Method, Cu^2+^-, Co^2+^- and Ni^2+^, ampicillin combinations have a highly synergistic antibiotic effect. Cd^2+^ and Zn^2+^-ampicillin interactions can be observed in SFigures 1E,F. Figures 3D,E show that Cu^2+^-, Co^2+^-kanamycin significant combinations are also synergistic comparing with the rest of the combinations which exhibit interactions either additive or antagonistic; such is the case of the Ni^2+^-kanamycin combinations (Figure 3F). Cd^2+^ and Zn^2+^-ampicillin interactions can be observed in SFigures 1G,H.

**Figure 4:**
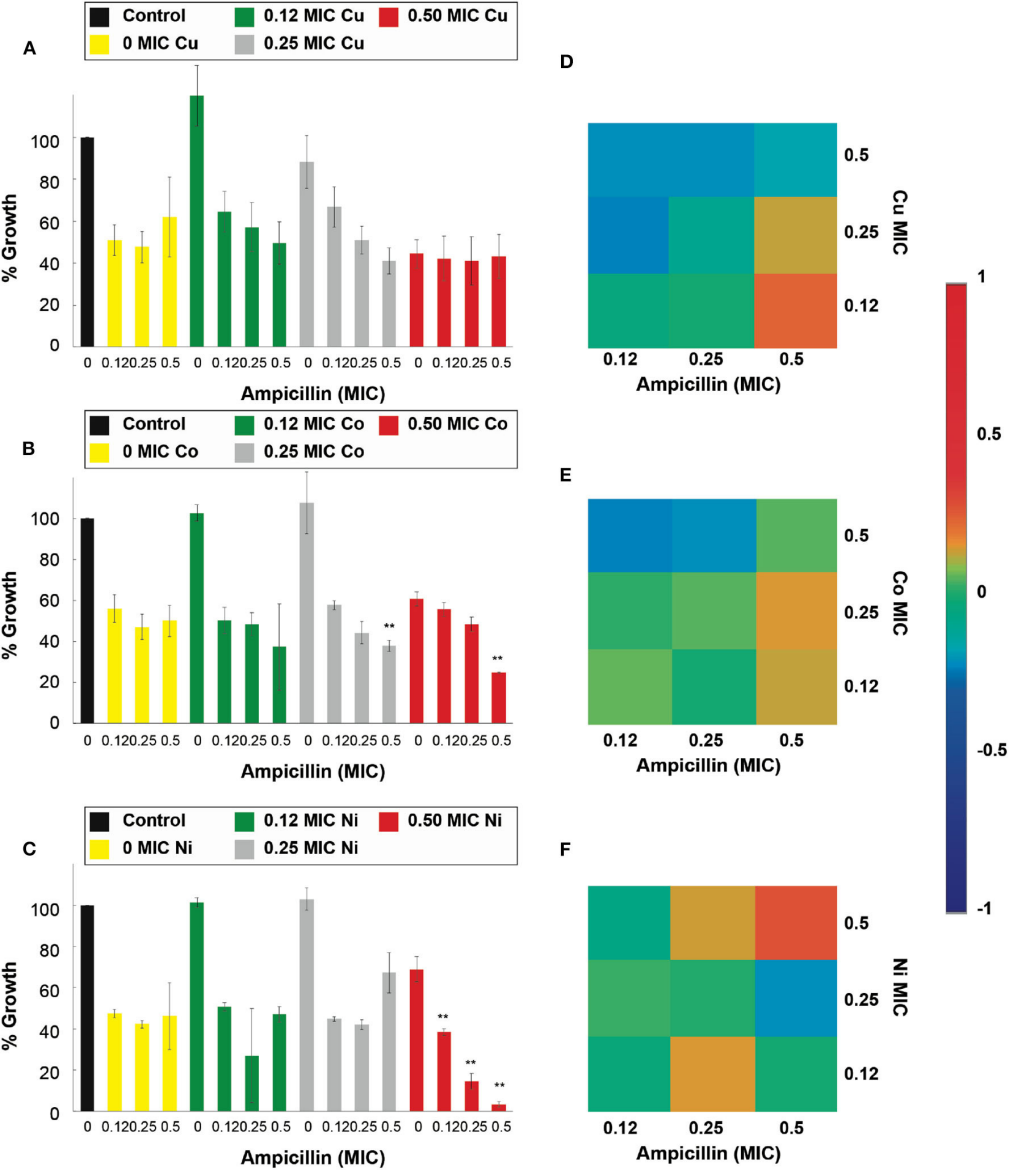
Antimicrobial effect and interactions of ATMCs in *S. aureus*. Growth percentage by sub-inhibitory concentrations of metal ion-antibiotic combinations: **(A)** Cu^2+^-ampicillin, **(B)** Co^2+^-ampicillin, and **(C)** Ni^2+^-ampicillin against *S. aureus* ATCC 6538. Classification of the different interactions between metal ion-antibiotic combinations. The interactions of **(D)** Cu^2+^-ampicillin, **(E)** Co^2+^-ampicillin, and **(F)** Ni^2+^-ampicillin combinations are classified as synergistic, additive or antagonist, value >0, =0, and <0, respectively. Each experiment was done in triplicates. **Corresponds to a significant difference (*p* < 0.05) concerning the control and each of the individual treatments. Error bars correspond to the standard deviation from experiments performed in triplicates.

Combinatory assays in *S. aureus* showed that only Ni^2+^ and Co^2+^ combined with ampicillin could exhibit a significant increase (*p* < 0.05) of the antibiotic inhibitory effect. Cu^2+^ could not enhance the antimicrobial effects of the antibiotic at the concentrations used in this work (Figure 4A). Co^2+^ at 0.5 and 0.25 of its MIC enhanced the effect of the 0.5 ampicillin MIC fraction (Figure 4B). Meanwhile, 0.5 Ni^2+^ MIC increased the effect of ampicillin when combined with all of the ampicillin MIC fractions (Figure 4C). Cd^2+^ and Zn^2+^ did show a significant increase in the inhibitory effect of ampicillin (SFigures 2A,B).

**Figure 5:**
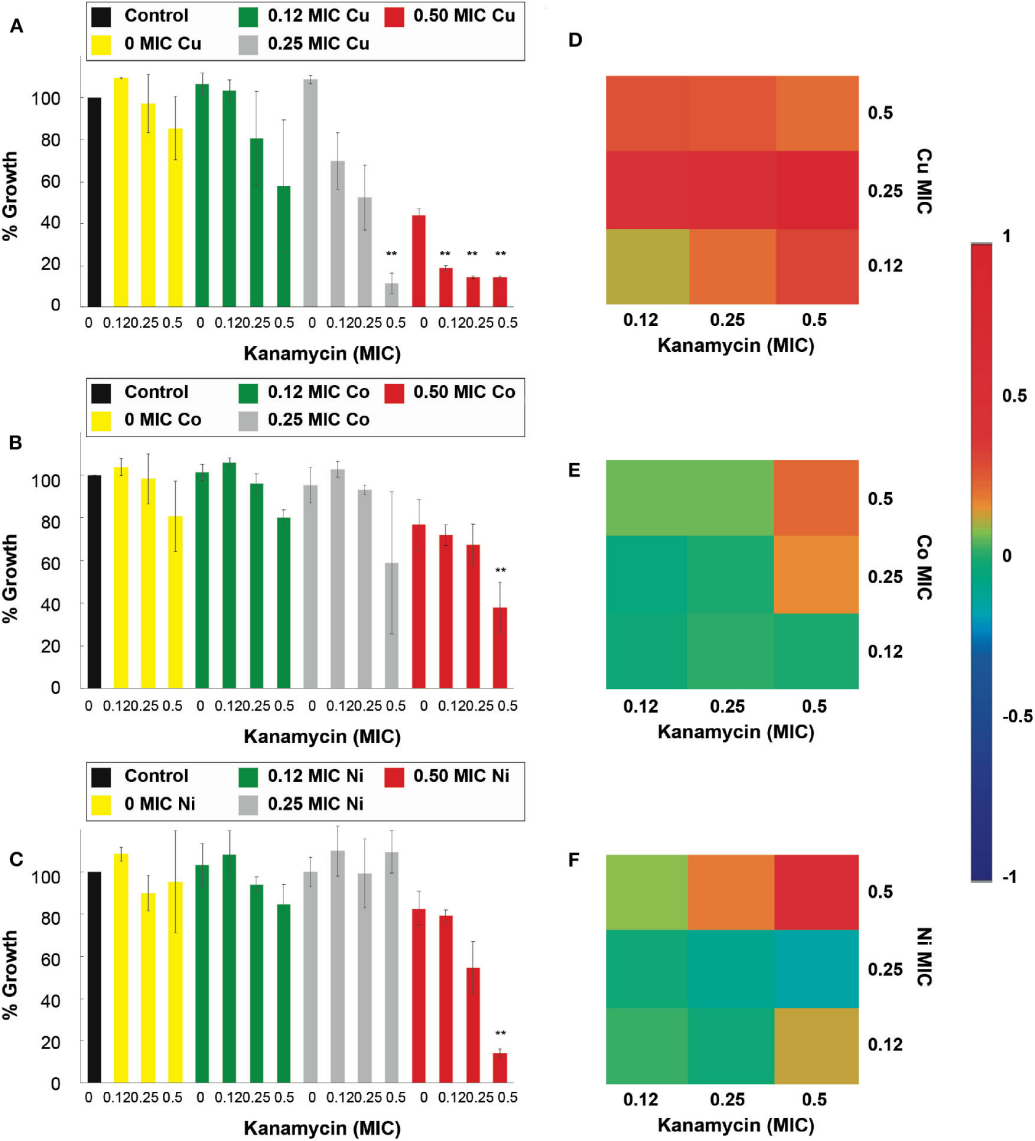
Antimicrobial effect and interactions of ATMCs in *S. aureus*. Growth percentage by sub-inhibitory concentrations of metal ion-antibiotic combinations: **(A)** Cu^2+^-kanamycin, **(B)** Co^2+^-kanamycin, and **(C)** Ni^2+^kanamycin against *S. aureus* ATCC 6538. Classification of the different interactions between metal ion-antibiotic combinations. The interactions of **(D)** Cu^2+^-kanamycin, **(E)** Co^2+^-kanamycin, and **(F)** Ni^2+^-kanamycin combinations are classified as synergistic, additive or antagonist, value >0, =0, and <0, respectively. Each experiment was done in triplicates. **Corresponds to a significant difference (*p* < 0.05) concerning the control and each of the individual treatments. Error bars correspond to the standard deviation from experiments performed in triplicates.

The results of the combinatorial treatments using kanamycin and the transition metal ion in *S. aureus* were different than those observed with ampicillin since all transition metal ions displayed an increased inhibitory effect. Figure 5A shows that 0.5 Cu^2+^ MIC was capable of significantly (*p* < 0.05), increase the effectiveness of kanamycin at all of the ranges of MICs tested; furthermore, at 0.25 Cu^2+^ MIC an increased effect on the 0.5 kanamycin MIC is also observed. Only 0.5 of the Co^2+^ and Ni^2+^ MIC with 0.5 kanamycin MIC had a significant potentiation effect (Figures 5B,C). Interestingly, at low concentrations of Zn^2+^ (0.12 MIC), a significant (*p* < 0.05) increase of the effect was observed for 0.5 kanamycin MIC (SFigure 2C). However, by increasing the Zn^2+^concentration in the combinations, the enhancement effect on kanamycin disappeared (SFigure 2C). For the case of the Cd^2+^ combinations, increased activity was observed for all Cd^2+^ MICs combined with 0.5 and 0.25 kanamycin MICs (SFigure 2D).

The nature of the potentiation effects of the combinatorial treatments was similar to the results observed in the treatments against *E. coli*. The ATMCs were synergistic at all the combinations where a significantly enhanced effect was observed; as can be seen in Figures 4D–F for Cu^2+^, Co^2+^, and Ni^2+^-ampicillin combinations, respectively, as well as in Figures 5D–F for Cu^2+^, Co^2+^, and Ni^2+^-kanamycin combinations, respectively. The nature of the interaction for Cd^2+^ and Zn^2+^-ampicillin, as well as for Cd^2+^ and Zn^2+^-kanamycin combinations, can be observed in SFigures 2E–H.

### Antimicrobial Effect of the ATMCs in Resistant Bacteria

In the data presented, even when most of the ATMCs show synergistic interactions, not all the transition metal ions used significantly enhanced the antimicrobial effect of the antibiotics in combination. Some ATMCs exhibited different inhibitory effects in the Gram-positive (*S. aureus*) and Gram-negative (*E. coli*) bacteria. We therefore, next, tested the antimicrobial effect of these ATMCs against the resistant bacterial strains, using the same methodology employed in the assays with the susceptible strains. With these assays, we explored the ability of the combinatorial treatments to overcome resistance mechanism and therefore re-sensitize bacterial strains to the antibiotic of resistance. The combinations used for these assays were at those concentrations that showed 80% growth inhibition in the sensitive bacteria. We observed that some transition metal ions, with significant inhibition in the ATMCs in the susceptible strains, continue being capable of enhancing the antimicrobial effect of the antibiotic in the resistant strains. In the assays with resistant *E. coli* strains, three transition metal ions (Cu^2+^, Ni^2+^, and Cd^2+^) re-sensitized *E. coli* to ampicillin and four transition metal ions (Cu^2+^, Co^2+^, Zn^2+^, and Cd^2+^) re-sensitized *E. coli* to kanamycin. Meanwhile, against the resistant *S. aureus* strains, three transition metal ions (Cu^2+^, Zn^2+^, and Cd^2+^) re-sensitized *S. aureus* to kanamycin, and no metal ion showed this capacity for ampicillin. Table 3 summarizes the significant potentiation of the ATMCs used in this work.

**Figure 6:**
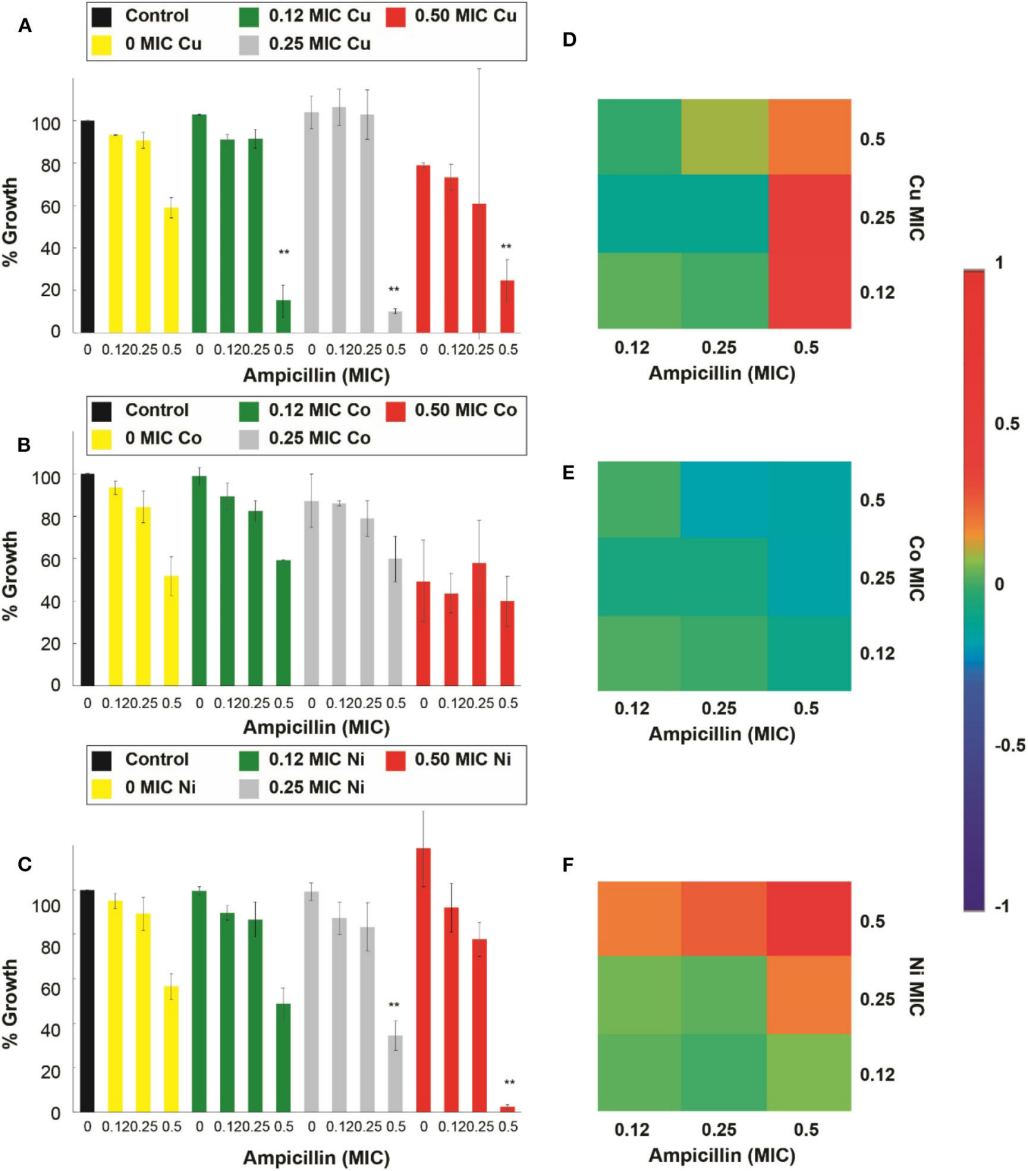
Antimicrobial effect and interactions of ATMCs in ampicillin-resistant *E. coli*. Growth percentage by sub-inhibitory concentrations of metal ion-antibiotic combinations: **(A)** Cu^2+^-ampicillin, **(B)** Co^2+^-ampicillin, and **(C)** Ni^2+^-ampicillin against *E. coli-Amp*. Classification of the different interactions between metal ion-antibiotic combinations. The interactions of **(D)** Cu^2+^-ampicillin, **(E)** Co^2+^-ampicillin, and **(F)** Ni^2+^-ampicillin combinations are classified as synergistic, additive or antagonist, value >0, =0 and, <0, respectively. Each experiment was done in triplicates. **Corresponds to a significant difference (*p* < 0.05) concerning the control and each of the individual treatments. Error bars correspond to the standard deviation from experiments performed in triplicates.

The results of the antimicrobial assays against *E. coli* resistant to ampicillin (*E. coli-Amp)* are shown in Figure 6 and it can be observed that Cu^2+^, Cd^2+^, and Ni^2+^, when combined with ampicillin, showed a significant (*p* < 0.05) inhibition of the resistant strain. Cu^2+^ increased the effect of the 0.5 MIC fraction of the antibiotic at all its MIC fractions (Figure 6A). However, unlike the positive results for the susceptible strains, Co^2+^ was not capable of exhibiting a potentiation capacity against the resistant strain (Figure 6B). The 0.5 Ni^2+^ MIC, similarly to the potentiation effect in susceptible *E. coli* assays, potentiated the effect of 0.5 ampicillin MIC (Figure 6C). The 0.5 and 0.25 Cd^2+^ MIC potentiated the ampicillin antimicrobial effect of the entire MIC range tested (SFigure 3A). Moreover, at 0.12 Cd^2+^ MIC, the inhibitory effect was observed at the 0.5 ampicillin MIC fraction. As observed with the susceptible strains, not all the transition metal ion could potentiate the effect of ampicillin in the resistant strain.

**Figure 7:**
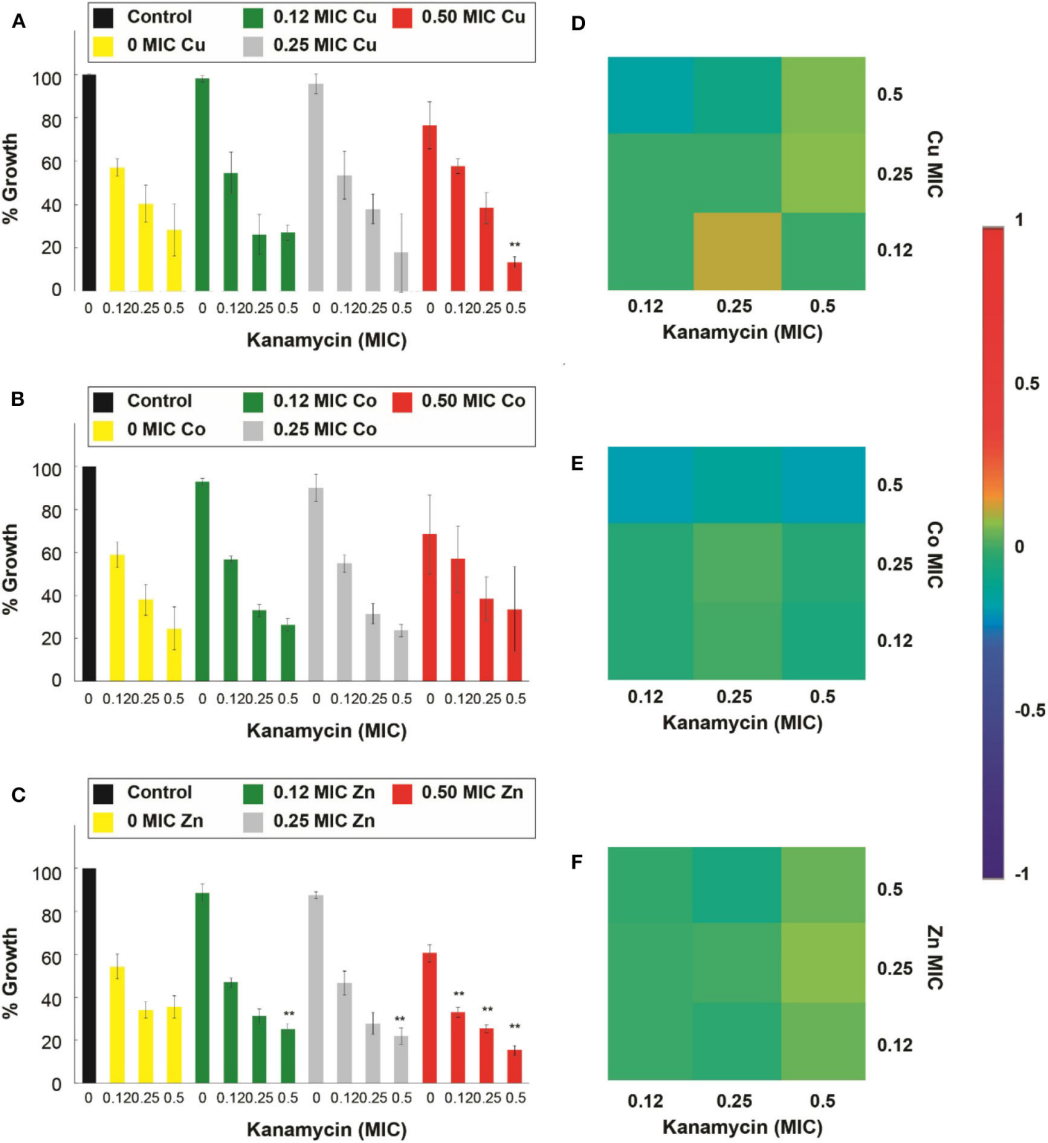
Antimicrobial effect and interactions of ATMCs in kanamycin-resistant *E. coli*. Growth percentage by sub-inhibitory concentrations of metal ion-antibiotic combinations: **(A)** Cu^2+^-kanamycin, **(B)** Co^2+^-kanamycin, and **(C)** Zn^2+^kanamycin against *E. coli-Kan*. Classification of the different interactions between metal ion-antibiotic combinations. The interactions of **(D)** Cu^2+^-kanamycin, **(E)** Co^2+^-kanamycin, and **(F)** Zn^2+^-kanamycin combinations are classified as synergistic, additive or antagonist, value >0, =0, and <0, respectively. Each experiment was done in triplicates. **Corresponds to a significant difference (*p* < 0.05) concerning the control and each of the individual treatments. Error bars correspond to the standard deviation from experiments performed in triplicates.

For the assays performed against the *E. coli* resistant to kanamycin (*E. coli-Kan)*, and shown in Figure 7, Cu^2+^, Zn^2+^, and Cd^2+^, enhanced significantly (*p* < 0.05) the kanamycin antimicrobial effect. Specifically, Cu^2+^, at 0.5 of its MIC, potentiated the activity of the 0.5 kanamycin MIC (Figure 7A). For the case of Co^2+^, none of the combinations showed an enhancement in the inhibitory effect of kanamycin at the concentrations used in this work (Figure 7B). For_Zn^2+^ all of its MIC fractions enhanced the 0.5 MIC kanamycin fraction. The 0.5 Zn^2+^ MIC fraction enhanced the 0.25 and 0.12 kanamycin MIC fractions (Figure 7C). All the kanamycin combinations with Cd^2+^ were enhanced, causing a significant inhibition (SFigure 3B).

Figure 6D demonstrates that all of the significant Cu^2+^-ampicillin combinations are found highly synergistic. However, all of the Co^2+^-ampicillin combinations were found antagonistic. The Ni^2+^-ampicillin combination with significant inhibition was found highly synergistic (Figure 6F), and most of the Cu^2+^ kanamycin combinations exhibit an additive nature (Figure 7D). However, most of the Co^2+^ combinations were found antagonistic (Figure 7E). For the case of the Zn^2+^-kanamycin combinations, the significant inhibitory combinations at 0.5 MIC of the antibiotic showed a synergistic nature, and even when some combinations are significantly inhibitory, their behavior was found to be additive (Figure 7F). The combinations of Cd^2+^ with both antibiotics can be observed in SFigures 3C,D.

**Figure 8:**
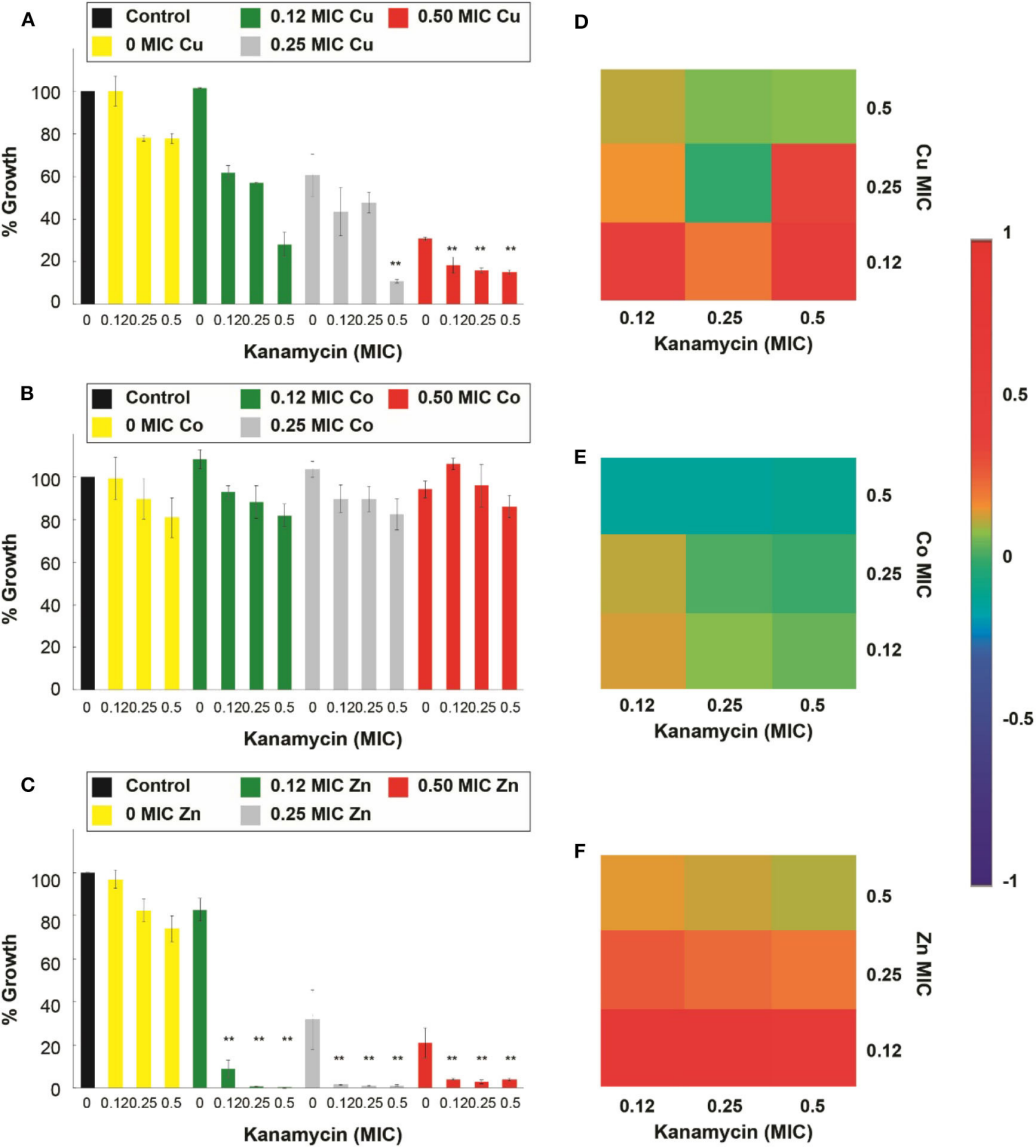
Antimicrobial effect and interactions of ATMCs in kanamycin-resistant *S. aureus*. Growth percentage by sub-inhibitory concentrations of metal ion-antibiotic combinations: **(A)** Cu^2+^-kanamycin, **(B)** Co^2+^-kanamycin, and **(C)** Zn^2+^kanamycin against *S. aureus-Kan*. Classification of the different interactions between metal ion-antibiotic combinations. The interactions of **(D)** Cu^2+^-kanamycin, **(E)** Co^2+^-kanamycin, and **(F)** Zn^2+^-kanamycin combinations are classified as synergistic, additive, or antagonist, value >0, =0, and <0, respectively. Each experiment was done in triplicates. **Corresponds to a significant difference (*p* < 0.05) concerning the control and each of the individual treatments. Error bars correspond to the standard deviation from experiments performed in triplicates.

Our subsequent antimicrobial assays were performed on an ampicillin-resistant *S. aureus* strain (*S. aureus-Amp)*, and the micronutrient transition metal ion tested was Ni^2+^ (SFigure 4A). However, even though the combination had shown synergistic interactions and the capacity to enhance the antimicrobial effect of ampicillin in the susceptible strain, the combination was not able to improve the effectiveness of the antibiotic in the tested resistant strain. Further, we explored antimicrobial assays in a kanamycin-resistant *S. aureus* strain (*S. aureus-Kan)*, and the results showed that for the case of Cu^2+^, Zn^2+^, and Cd^2+^ there was a significant (*p* < 0.05) increase in the antimicrobial effect of kanamycin in the combinatorial treatment. Cu^2+^, at 0.5 of its MIC, improved all the kanamycin MIC fractions (Figure 8A); moreover, kanamycin at 0.5 MIC fraction was also enhanced at 0.25 Cu^2+^ MIC. Moreover, the addition of Co^2+^ did not have a positive effect on the kanamycin antimicrobial effect (Figure 8B). For the case of Zn^2+^, all the MIC fractions tested potentiated all the kanamycin MIC fractions (Figure 8C). The assays involving antibiotic combinations with Cd^2+^ revealed that the transition metal ion improved the effect of all the kanamycin MIC fractions at 0.5 and 0.25 Cd^2+^ MIC (SFigure 4B) significantly. Additionally, the Cd^2+^ 0.5 MIC fraction combinations were not significantly different from the controls (antibiotic and metal ion).

The interactions of the antibiotic with the micronutrient transition metal ions tested, at the combinations that significantly enhanced the antimicrobial properties of the antibiotic, according to the Bliss Method, represent a synergistic behavior; such is the case of Cu^2+^- and Zn^2+^-kanamycin (Figures 8D–F). Additionally, the results also show that there are cases where even when the interaction is lowly synergistic, the inhibition was not significantly different from the antimicrobial agents used separately; such is the case for Co^2+^-kanamycin (Figure 8E), Ni^2+^-ampicillin and Cd^2+^-kanamycin interactions (SFigures 4C,D).

### *In vivo* Toxicological and Antimicrobial Study

**Figure 9:**
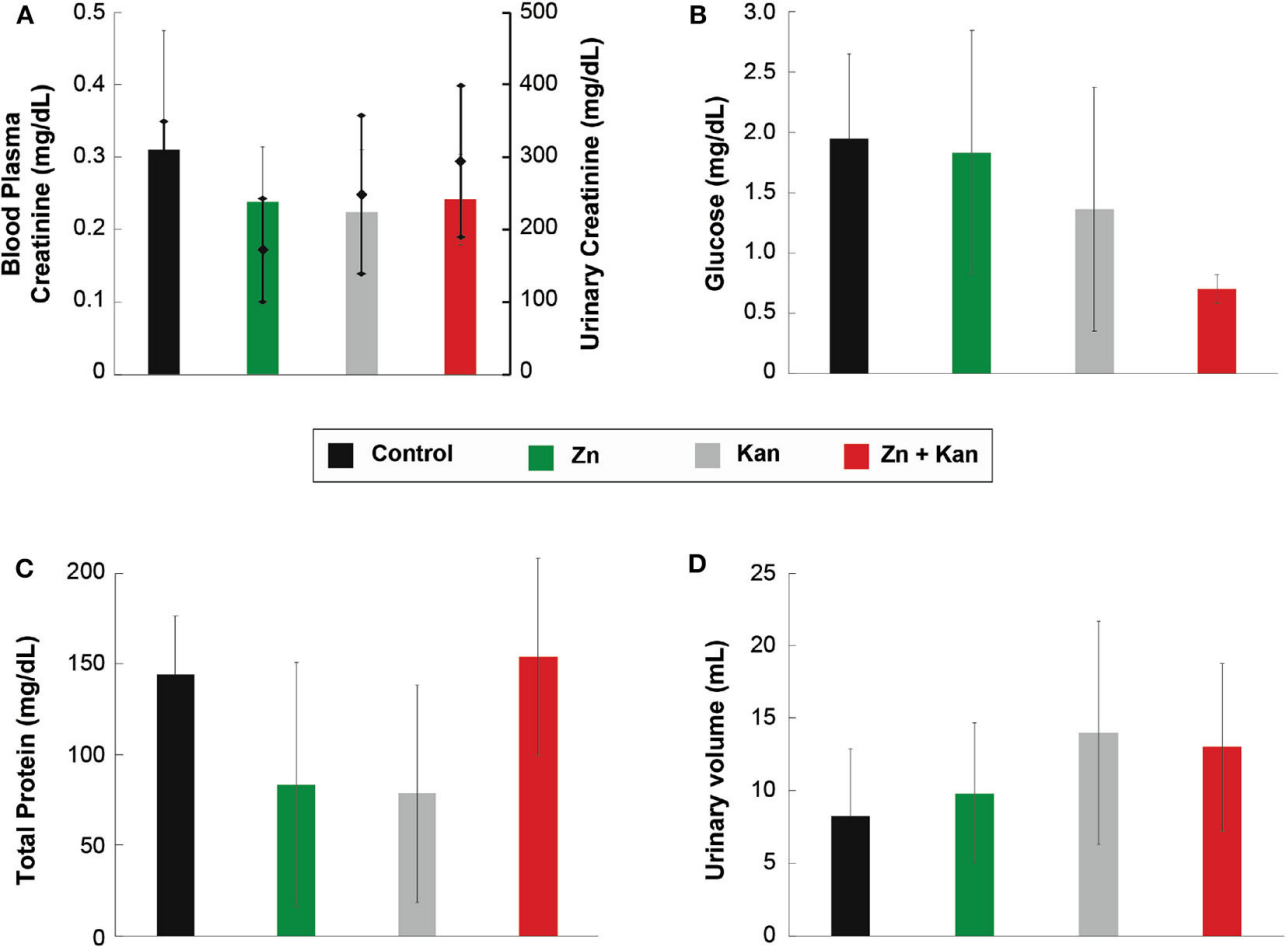
Effect of Zn-kanamycin combination on the function of kidney in male Wistar rats. **(A)** Urinary (diamonds) and blood plasma (bars) creatinine levels. **(B)** Urinary glucose levels. **(C)** Total urine proteins. **(D)** Urinary volume. All experiments were tested in groups of *n* = 5 for 3 days. Error bars represent ± SD.

**Figure 10:**
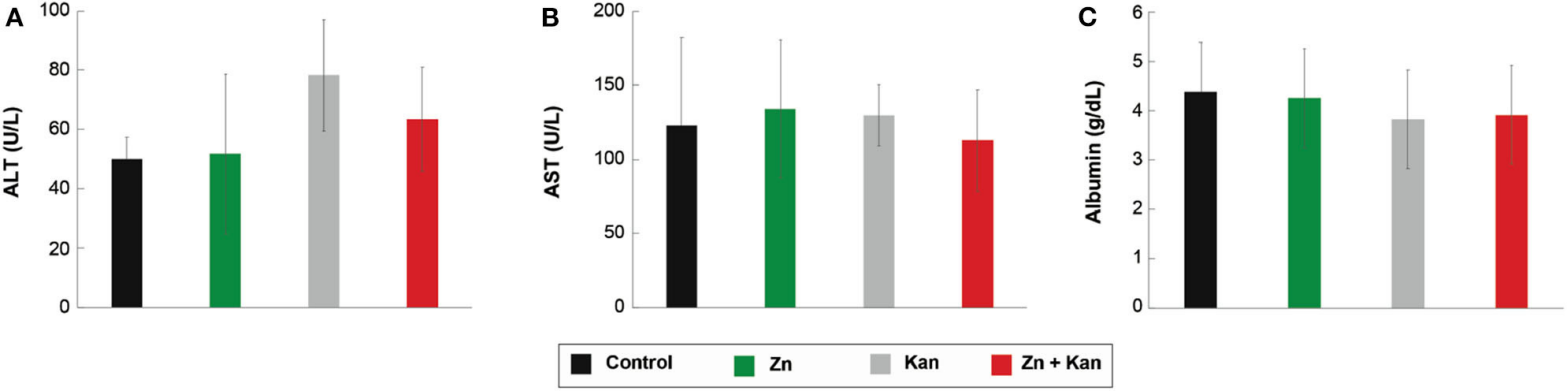
Effect of Zn-kanamycin combination on the function of the liver in male Wistar rats. **(A)** ALT, **(B)** AST, and **(C)** Albumin levels. All experiments were tested in groups of *n* = 5 for 3 days. Error bars represent ± SD.

**Figure 11:**
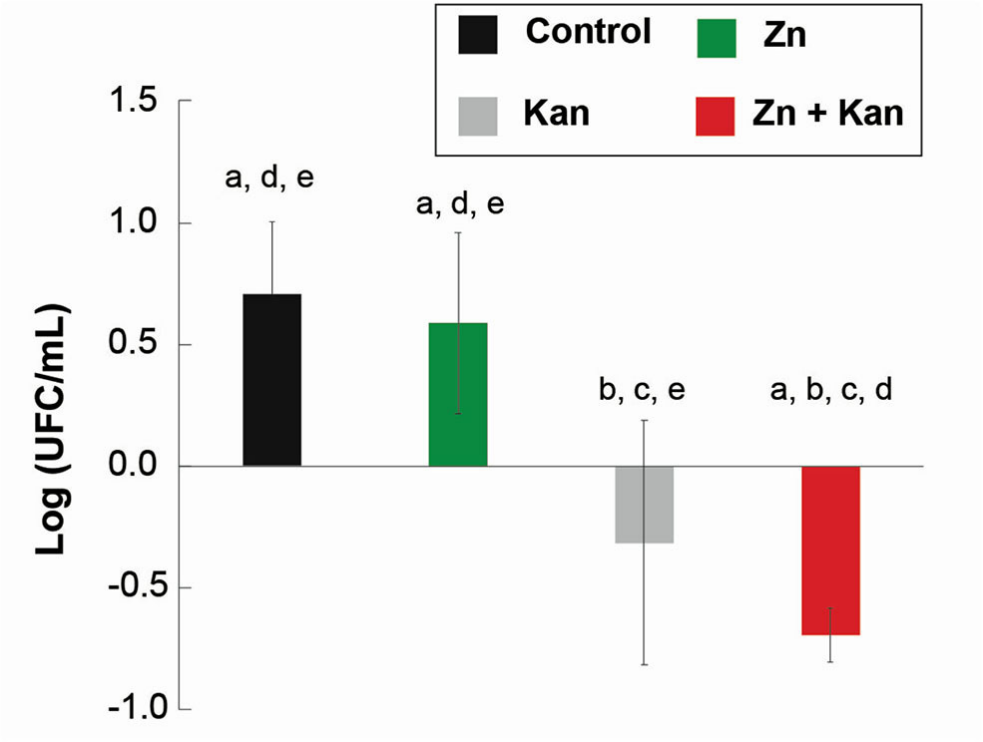
*In vivo* topical antimicrobial effect of Zn-kanamycin combination in male Wistar rats. Differences from the uninfected group (baseline) of the bactericidal effect of Zn, kanamycin, and Zn-kanamycin combination (all at 0.5 MIC) in *S. aureus-Kan* topical infection growth. Letters above the bars indicate a significant difference (*p* < 0.05) from a: uninfected, b: infected, c: Zn, d: kanamycin, and e: Zn- kanamycin groups. All experiments were tested in groups of *n* = 3 for 3 days. Error bars represent ± SD.

**Figure 12:**
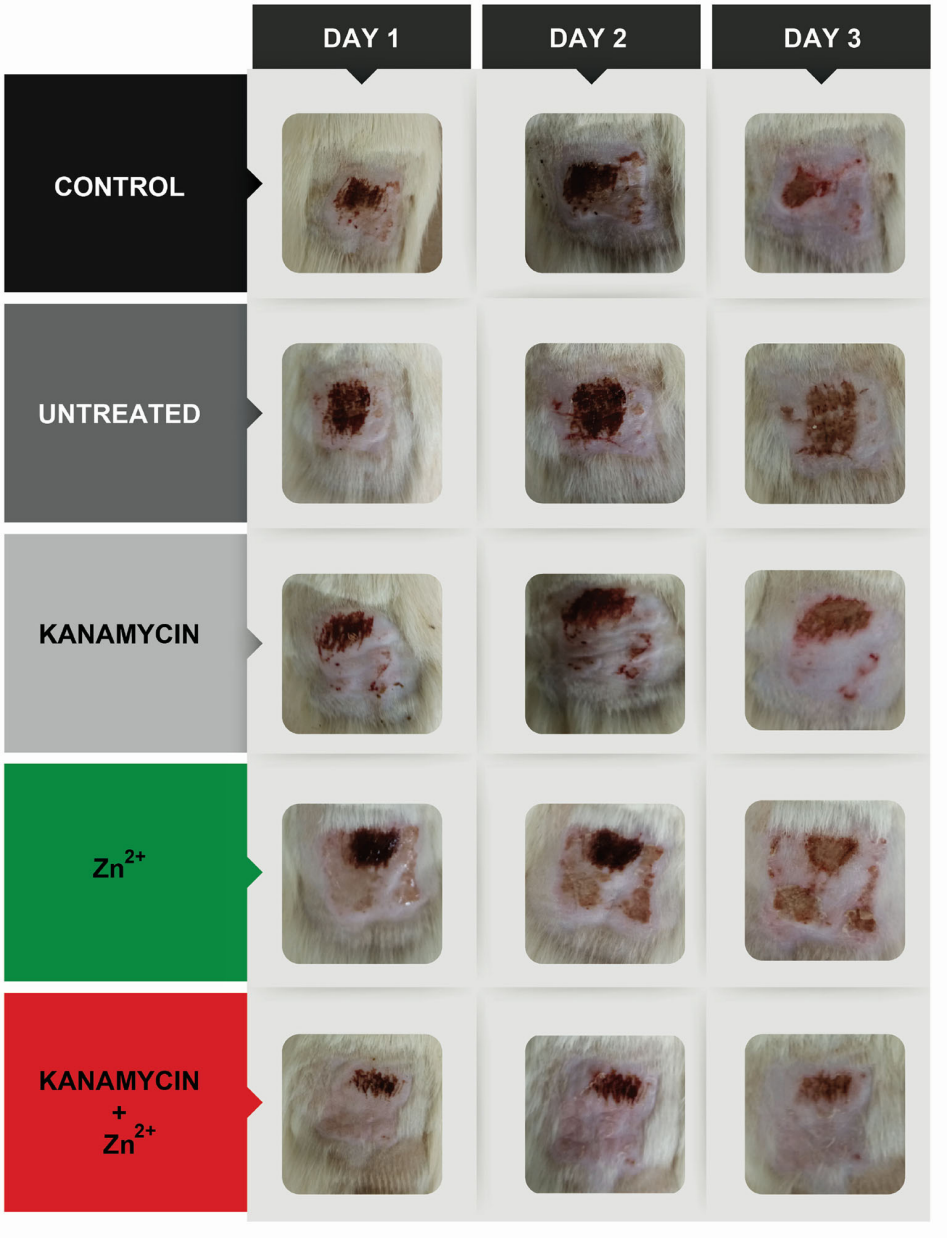
*In vivo* topical infection model with *S. aureus-Kan* induced by a tape stripping model. Images show the evolution of the *in vivo* infection after 3 days in the control, an untreated infection and infections treated with Zn, kanamycin, and Zn-kanamycin combinations (all at 0.5 MIC).

Due to the large number of significant interactions (compared with the low toxic transition metal ion) and the reduction of its toxicity against HaCat cells when used in combination, we tested Zn^2+^, kanamycin and Zn^2+^-kanamycin combination, in an *in-vivo* model, at a concentration capable to re-sensitize kanamycin-resistant *S. aureus*. As can be observed in Figures 9A–D, all the measured renal parameters showed no significant differences between the controls and the treated groups. Furthermore, this work analyzed several hepatic function parameters in blood plasma. ALT, AST, and albumin concentrations were measured as biomarkers of hepatic cell necrosis, damage, and hepatic function of the treated groups with Zn^2+^/kanamycin. Similar to the obtained results for renal function, there were no significant differences between the treated and the untreated control groups in the parameters linked to determine the hepatic function (Figures 10A–C). For the antimicrobial *in vivo* assay, a topical infection with the *S. aureus-Kan* strain was made. We observed that Zn^2+^-kanamycin combinations were capable of significantly decreasing the resistant bacterial load (*p* > 0.05), as can be seen from Figure 11. Moreover, the topical infection induced by the tape stripping model, was monitored for 3 days, after initiating treatment with the different compounds; and the results can be observed in Figure 12.

## Discussion

### Antimicrobial Activity of Selected Metal Ions and Antibiotics

It has been well-documented that the use of metal ions to take advantage of their antimicrobial properties has been a practice widely used by society since the first civilizations (Magner, 1992; Rai et al., 2009; Grass et al., 2011). The MICs of the metal ion obtained in this work, Table 1, are congruent with those previously reported in the literature for Zn^2+^(Beard et al., 1997), Ni^2+^ (Brocklehurst and Morby, 2000) Co^2+^ and Cd^2+^ (Spada et al., 2002). Even though the MICs reported are similar to those previously described, some small discrepancies can be associated with differences in the culture media used (Brocklehurst and Morby, 2000; Lee et al., 2005). Similarly, Table 2 summarizes the increase in MIC concentrations, expected due to the transformation of the bacteria strains with a plasmid vector containing specific antibiotic resistance.

### Toxicity Data for the Different Transition Metal Ions

One of the big challenges associated with the use of transition metal ions, and any other compound, as antimicrobial agents, are their native toxicity to cells, eukaryotes, and prokaryotes. Toxicity assays must be done to ensure their safe used in a particular application. In this study, we suggest the use of combinatorial treatments, transition metal ions-antibiotics, as an alternative to decrease the cytotoxicity of the use of transition metal ions alone as antimicrobial agents. This strategy allows lowering the overall MIC, resulting in the design of an effective therapy below toxic concentrations.

Our research group has previously reported *in vitro* cytotoxicity analysis of different transition metal ions in two mammalian cell lines, rat cardiomyoblast cells to reference a systemic effect, and human keratinocytes cells to measure a local, topic effect (Garza-Cervantes et al., 2017). Previous studies (Garza-Cervantes et al., 2017) showed the potential use of these transition metal ions as topical antimicrobial agents. Therefore, in this work, we focused on a final topical treatment as an application. The topical toxicity effects in HaCat cells are observed at concentrations of 4, 2, 2, 1, and 2 mM for Cu^2+^, Zn^2+^, Cd^2+^, Co^2+^, and Ni^2+^, respectively. In this research, we worked with the *E. coli* MIC at 8, 2, 1, 1, and 2 mM for Cu^2+^, Zn^2+^, Cd^2+^, Co^2+^, and Ni^2+^, respectively. Most of these concentrations are either close or slightly lower than the toxic concentrations for HaCat cells. However, when testing combinations with the antibiotics, the results show that several fractions reduced the MICs. Thus, the antimicrobial concentrations of the transition metal ions are here significantly reduced to 1, 0.25, 0.125, 0.125, and 0.25 MIC for Cu^2+^, Zn^2+^, Cd^2+^, Co^2+^, and Ni^2+^, respectively, when they are combined with the different antibiotics.

Based on these results the toxicity of transition metal ion, and the antibiotic treatments alone, as well as the toxicity of the combinatorial treatments were explored in HaCat cells. Most of the transition metal ions tested (Cu^2+^, Ni^2+^, and Zn^2+^) exhibited antimicrobial effects when combined with the antibiotics at non-toxic concentrations for human keratinocyte cells, as expected due to the use of the sub-inhibitory concentrations required for this work, even at the concentrations tested against the resistant bacteria. We also observed a decreased toxicity in the Zn^2+^-kanamycin combinations when used at a sub-inhibitory concentration of the resistant bacteria. These observed results, where the combinatorial treatments decreased the toxicity threshold of metal ion, could be due to the interactions between the metal ions and the antibiotics. It has been reported that kanamycin can form complexes with transition metal ions like Cu^2+^, and Zn^2+^ (Mashaly, 1993) and therefore suggest that the Zn^2+^-kanamycin interactions are decreasing the toxicity effects of Zn^2+^ on mammalian cells without disrupting their antimicrobial effect (Szczepanik et al., 2004; Shi et al., 2008).

### Antimicrobial Effect of Antibiotic/Micronutrient Transition Metal Ion Combinations

Antibiotic resistance has become a global problem that has increased in the last decade, leading researches to investigate how to overcome this issue. One of the strategies adopted is the use of adjuvant components focused on retarding the emergence of antibiotic-resistant and the possibility of obtaining synergistic combinations (Lima et al., 2013). Here, we propose the use of transition metal ions as enhancers of commonly used antibiotics -ampicillin and kanamycin- against both susceptible and resistant, *E. coli* and *S. aureus*.

Once we determined the MICs of each antibiotic and transition metal ion, we proceeded to test the ATMCs through a checkerboard methodology. This methodology is widely used to assess the capacity of two antimicrobial agents to enhance the antimicrobial effect of each other when used in combination (Hsieh et al., 1993; Bajaksouzian et al., 1997; Sweeney and Zurenko, 2003; Orhan et al., 2005). We tested sublethal concentrations of ampicillin (β-lactam antibiotic) and kanamycin (aminoglycoside antibiotic) combined with a sublethal concentration of each of the transition metal ions, Cu^2+^, Co^2+^, Ni^2+^, Zn^2+^, and Cd^2+^, against *E. coli* and *S. aureus* strains. The results showed significantly enhanced antimicrobial activity. We applied the Bliss Independence model to determine the nature of the antimicrobial effect exhibited by the ATMCs and quantified the degree of synergism between the transition metal ion and each of the antibiotics (Hegreness et al., 2008).

Through *in vitro* antimicrobial assays, we demonstrated that Cu^2+^, Co^2+^, Cd^2+^, Ni^2+^, and Zn^2+^, in correlation with the results obtained in this work, are capable of enhancing the inhibitory effect of the β-lactam antibiotic ampicillin and the aminoglycoside antibiotic kanamycin at sub-inhibitory concentrations against *E. coli* and *S. aureus*; under the conditions implemented in this study. Though, the extent to which each ATMC affected the antimicrobial effect of the antibiotics differed significantly between bacterial species. Cu^2+^, Co^2+^, Cd^2+^, and Ni^2+^ enhanced synergistically the antimicrobial effect of ampicillin against susceptible *E. coli* meanwhile Ni^2+^ enhanced the effect of this antibiotic against susceptible *S. aureus*. When used in combination with the aminoglycoside antibiotic, kanamycin, Cu^2+^, Co^2+^, Cd^2+^, and Zn^2+^ enhanced its antimicrobial kanamycin against susceptible *E. coli*; as well as Cu^2+^, Ni^2+^, Cd^2+^, and Zn^2+^ against susceptible *S. aureus*.

Different authors have reported studies focused on the use of transition metal ions in combination with antibiotics (El-Gamel, 2010; Selvaraj et al., 2019; Falcón García et al., 2020) and in general the results showed positive effects (Bagihalli et al., 2008). However, there have been reports that show that some antibiotics-metal ion concentrations can have antagonistic interactions between each other (Tommasino et al., 2011). We observed these phenomena in our results. For the combinations tested in *E. coli*, when combining Zn^2+^ with ampicillin, no increase of the inhibitory effect was notable, as well as when combining Ni^2+^ with kanamycin. However, there was a significant inhibitory effect when combining Ni^2+^ with ampicillin and Zn^2+^ with kanamycin. In *S. aureus* assays, only Ni^2+^ and Co^2+^ could increase the inhibitory effect of the β-lactam antibiotic, however, for the case of kanamycin, all the combinations with the transition metal ion showed significant potentiation at least at one of the MICs. The results demonstrate that the nature of the interactions in the combinations tends to be synergistic, at the high MIC fractions, in both bacterial strains. However, there are some cases where our results show antagonistic effects of the combinations for one strain and synergistic in the other, which is an effect that has also been observed previously in the literature for other combinatorial treatments (Chohan et al., 2004).

### Antimicrobial Effect of the ATMCs in Resistant Bacteria

Antibiotic resistance increases day-by-day, leading to an extensive search for solutions overcoming this worldwide problematic. Our data shows that when used in combination with antibiotics (ampicillin and kanamycin), some micronutrient transition metal ions have a synergistic antimicrobial effect against resistant Gram-negative and Gram-positive strains. When used in combination with ampicillin, Cu^2+^, Cd^2+^, and Ni^2+^, re-sensitized resistant *E. coli-Amp* strain; meanwhile Cu^2+^, Cd^2+^, and Zn^2+^ re-sensitized both resistant *E. coli-Kan* and *S. aureus-Kan* strains. On the other hand, Co^2+^ was not able to potentiate the antimicrobial effect of these antibiotics, in the resistant strains used in this study, even when it could potentiate the effect in the susceptible strains. Similarly, Ni^2+^ couldn't enhance the antimicrobial effect of ampicillin against resistant *S. aureus-Amp*.

Furthermore, our results show that some of the transition metal ions have different effects when combined with the antibiotics and tested against the resistant strains. There is a differential effect between strains of the same bacterial species, which has also been reported in the literature (Ruparelia et al., 2008). In addition, some Cu^2+^-ampicillin and Zn^2+^-kanamycin combinations enhanced the antibiotic activity at 0.12 and 0.5 MIC but not at 0.25 MIC. This kind of behavior is similar to previously reported data where checkerboard methodology has been used (Brochmann et al., 2016; Courtney et al., 2017; Garza-Cervantes et al., 2017; El Zahed and Brown, 2018). This response could be observed due to a non-linear dosage-response activity of the antimicrobial agent's combinations and their response in a complex matrix like bacterial/cellular cultures. Some synergy tests assume that all antimicrobial agents have a linear dosage-respond curve, like the FIC index and the isobologram interpretation (Pillai et al., 2005), but using a complete checkerboard assay and interpretation of synergy, such as the methods used in this work, increase the possibility of finding synergistic antimicrobial combinations.

As mentioned previously, the problem of antibiotic resistance has increased over the years. One of the potential pathways to combat antibiotic resistance is to explore the possibilities of bringing back inactive/older antibiotics (Nguyen, 2012). A strategy to overcome the problems caused by resistant bacteria is to be capable of inhibiting the expression of the resistant mechanisms of the microorganism, and this could bring back the use of conventional antibiotics which are less effective due to this problematic (Taylor et al., 2002; Marks et al., 2013). Different authors have reported the use of adjuvants to decrease the MIC of resistant bacteria using antibiotics against different Gram-positive and Gram-negative bacteria (Kim et al., 2018; Baker et al., 2019). These authors determined that the sensitizer effect observed for these compounds can be explained with their capacity to alter the stability/function of the outer or cytoplasmic membrane (Cadelis et al., 2019; French et al., 2019). Such is the case for transition metal ions, included the ones used in this work, which have been reported to disrupt these cellular structures including the outer and the cytoplasmic membrane (Lemire et al., 2013). Therefore, the transition metal ions used in this work are expected to be interacting with the bacterial membrane, as it contains a variety of polymers and proteins with electronegative chemical groups capable of coordinating with transition metal ions. These transition metal ions interact with the bacterial cell wall, increasing its permeability (Garza-Cervantes et al., 2017) in a way that allows more antibiotics to pass through surpassing the activity of the enzymes in charge of the resistance mechanisms. In addition, transition metal ions like Cu^2+^, Ni^2+^, and Zn^2+^, have been reported to cause not only membrane impairment, by reacting with proteins in the bacterial cell membrane, but also disruption of internal cellular processes that produce reactive oxygen species and lead to DNA alterations. (Hong et al., 2012; Lemire et al., 2013; Meghana et al., 2015; Dogra et al., 2018). The results in this work, therefore, suggest that the transition metal ions used in the formulations of the combinatorial treatments, could interact with the cell membrane, causing an increase in permeability, and allowing the entrance of both antibiotic and metal ions into bacteria. Finally, metal ions would interrupt internal processes leading to an increased effect of the antibiotic activity, resulting in enhanced bacterial inhibition of the combinatorial treatments.

Infections caused by *staphylococcus* are among the most common infections in hospitals, and *staphylococcus* is one of the most common genera present in skin infections (Kim et al., 2001; Stulberg et al., 2002; WHO, 2015). Therefore, since the combinatorial treatments reported in this work are intended to be used in future clinical treatments, we designed an experiment focused on testing the antimicrobial activity of an ATMC against *S. aureus-Kan in vivo* in a murine skin infection model. For this assay, we selected the treatment that combined Zn-kanamycin, since firstly, Zn^2+^ is a therapeutic agent widely used in the treatment of skin infections (Gupta et al., 2014). And secondly, in our results, Zn^2+^, in combination with kanamycin, showed that most of the combinations tested showed an enhancement in the antimicrobial effect against *S. aureus-Kan*. This in addition to the fact that our results show that toxicity of Zn^2+^ decreases for mammalian cells when combined with kanamycin. Therefore, we hypothesized that Zn-kanamycin combinatorial therapy could be useful to treat skin infections caused by a resistant *S. aureus*.

### *In vivo* Toxicological and Antimicrobial Study

For an organism to maintain a healthy balance of its internal environment, the correct functioning of its organs is needed. This balance is mostly achieved with the proper functioning of organs like the kidney and the liver. Therefore, renal function parameters in urine, total volume (amount of body fluid), glucose, total proteins, and creatinine (urine and plasma) concentration were assessed as indicators of tubular function, glomerular filtration, and general function of nephrons in the organism. An *in vivo* assay was performed with Zn^2+^-kanamycin combination capable to re-sensitize *S. aureus-Kan*. Our results showed no toxicological effects found in rat's kidney and liver parameters, however, and more interestingly for these non-toxic concentrations, the data displays a significant reduction of the CFU in the *S. aureus-Kan* infection when using the Zn^2+^-kanamycin combinations at 0.5 MIC of each. These results confirm the re-sensitization of the resistant strain and it shows that these phenomena are also exhibited in an *in vivo* infection setting. Zn^2+^ has been used for a wide range of dermatological applications, in concentrations up to 10–20% as zinc sulfate (Gupta et al., 2014). Previous data have reported a decrease of *S. aureus* surface attachment (Akiyama et al., 1998) and quantity of *Propionibacterium acnes* (Strauss and Stranieri, 1984), using Zn^2+^ formulations at 5 and 1.2%, respectively. Here we used a concentration of Zn^2+^ 4 mM (0.026%) as a topical treatment, and no toxicological effects were found.

Based on this data, we can conclude that these ATMCs could be applied in dermic therapeutic applications, although further investigation is needed regarding non-toxic concentrations for all of the ATMCs performed in this study. Altogether, our results suggest that, due to the inherent complexity of the synergistic effects observed in this study, this work provides a platform to focus further research on deciphering their antimicrobial mechanisms of action but also taking into account their relevance in the future design of novel therapeutic strategies.

## Data Availability Statement

All datasets generated for this study are included in the article/Supplementary Material.

## Ethics Statement

The animal study was reviewed and approved by ethics committee of the animal research facility at the Universidad Autónoma de Aguascalientes. The Animal Care and Use Committee of the Universidad Autónoma de Aguascalientes, adhering to the Official Mexican Regulations (NOM-062-ZOO-1999), evaluated the animal care and experimentation practices and approved protocol 25698-UANL-UAA-2019 to perform the experiments. Mexican regulations are in strict accordance with the recommendations in the Guide for the Care and Use of Laboratory Animals of the NIH and the Weatherall Report in the USA to ensure compliance with established international regulations and guidelines. At the end of experiments, animals were sacrificed by using an excess of sodium pentobarbital anesthesia (40 mg/kg bw). Efforts were made to minimize suffering.

## Author Contributions

JG-C, JM-B, HR-H, IS-C, OO-R, ES, CE-G, and JM-R designed, performed, and analyzed all the experimental data and wrote the manuscript. All authors contributed to the article and approved the submitted version.

## Conflict of Interest

The authors declare that the research was conducted in the absence of any commercial or financial relationships that could be construed as a potential conflict of interest.
