# Supplementary material for: Re-sensitizing Ampicillin and Kanamycin-Resistant E. coli and S. aureus Using Synergistic Metal Micronutrients-Antibiotic Combinations
Source: Front Bioeng Biotechnol. 2020 Jun 24;8:612. doi: 10.3389/fbioe.2020.00612 (PMC7327704; doi:10.3389/fbioe.2020.00612)
Supplement: Supplementary file 1 [file Table_1.DOCX]

Supplementary Materials
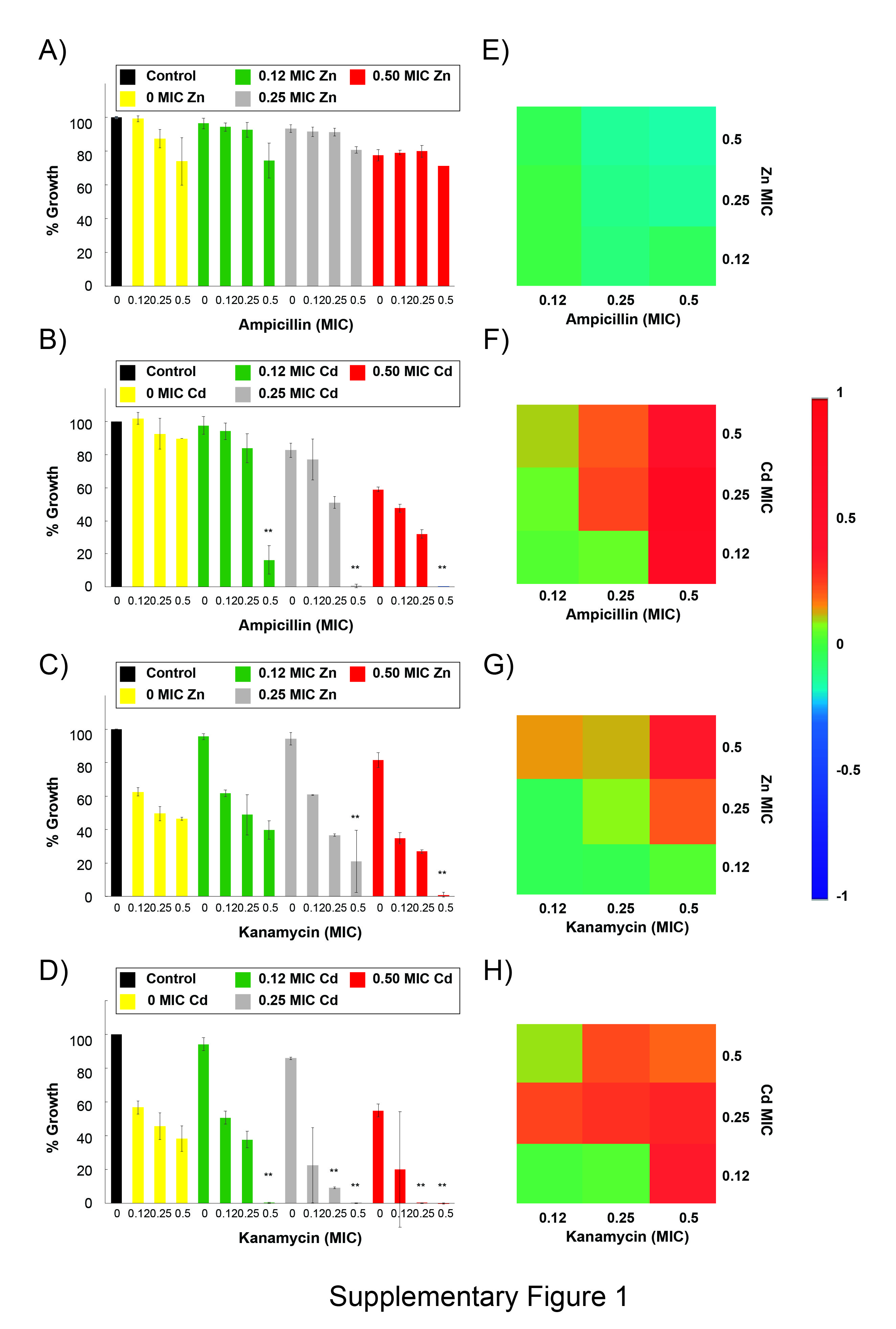


**SFigure 1.** Antimicrobial effect and interactions of ATMCs in *E. coli*. Growth percentage by sub-inhibitory concentrations of metal-antibiotic combinations: (**A**) Zn^2+^-ampicillin, (**B**) Cd^2+^-ampicillin, (**C**) Zn^2+^-kanamycin and (**D**) Cd^2+^-kanamycin against *E. coli* ATCC 11229. Classification of the different interactions between metal-antibiotic combinations. The interactions of (**E**) Zn^2+^-ampicillin, (**F**) Cd^2+^-ampicillin, (**G**) Zn^2+^-kanamycin and (**H**) Cd^2+^-kanamycin combinations are classified as synergistic, additive or antagonist, value >0, =0 and <0, respectively. Each experiment was done in triplicates. **Corresponds to a significant difference (p<0.05) with respect to the control and each of the individual treatments. Error bars corresponds to the standard deviation from experiments performed in triplicates.


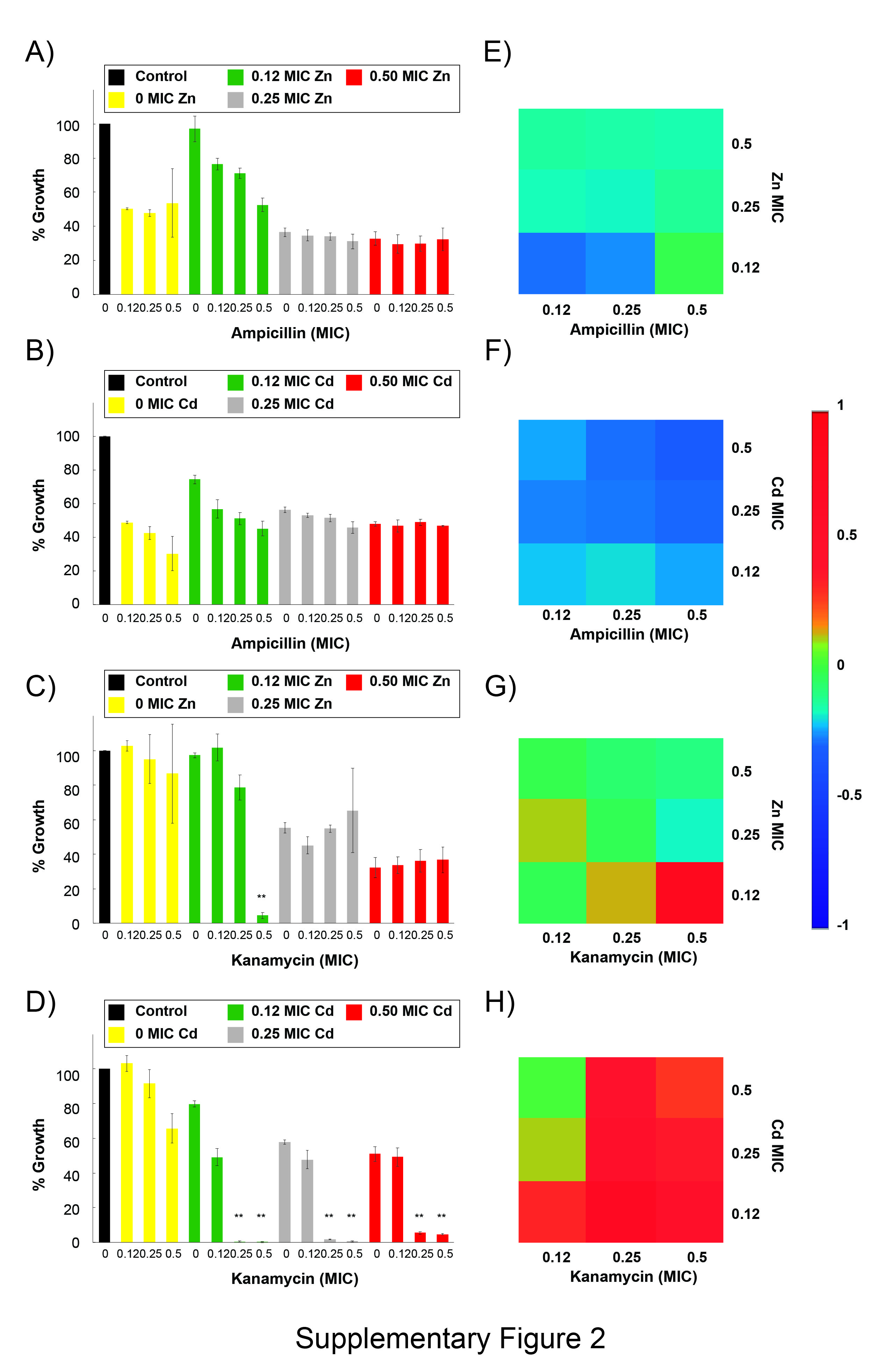


**SFigure 2.** Antimicrobial effect and interactions of ATMCs in *S. aureus*. Growth percentage by sub-inhibitory concentrations of metal-antibiotic combinations: (**A**) Zn^2+^-ampicillin, (**B**) Cd^2+^-ampicillin, (**C**) Zn^2+^-kanamycin and (**D**) Cd^2+^-kanamycin against *S. aureus* ATCC 6538. Classification of the different interactions between metal-antibiotic combinations. The interactions of (**E**) Zn^2+^-ampicillin, (**F**) Cd^2+^-ampicillin, (**G**) Zn^2+^-kanamycin and (**H**) Cd^2+^-kanamycin combinations are classified as synergistic, additive or antagonist, value >0, =0 and <0, respectively. Each experiment was done in triplicates. **Corresponds to a significant difference (p<0.05) with respect to the control and each of the individual treatments. Error bars corresponds to the standard deviation from experiments performed in triplicates.


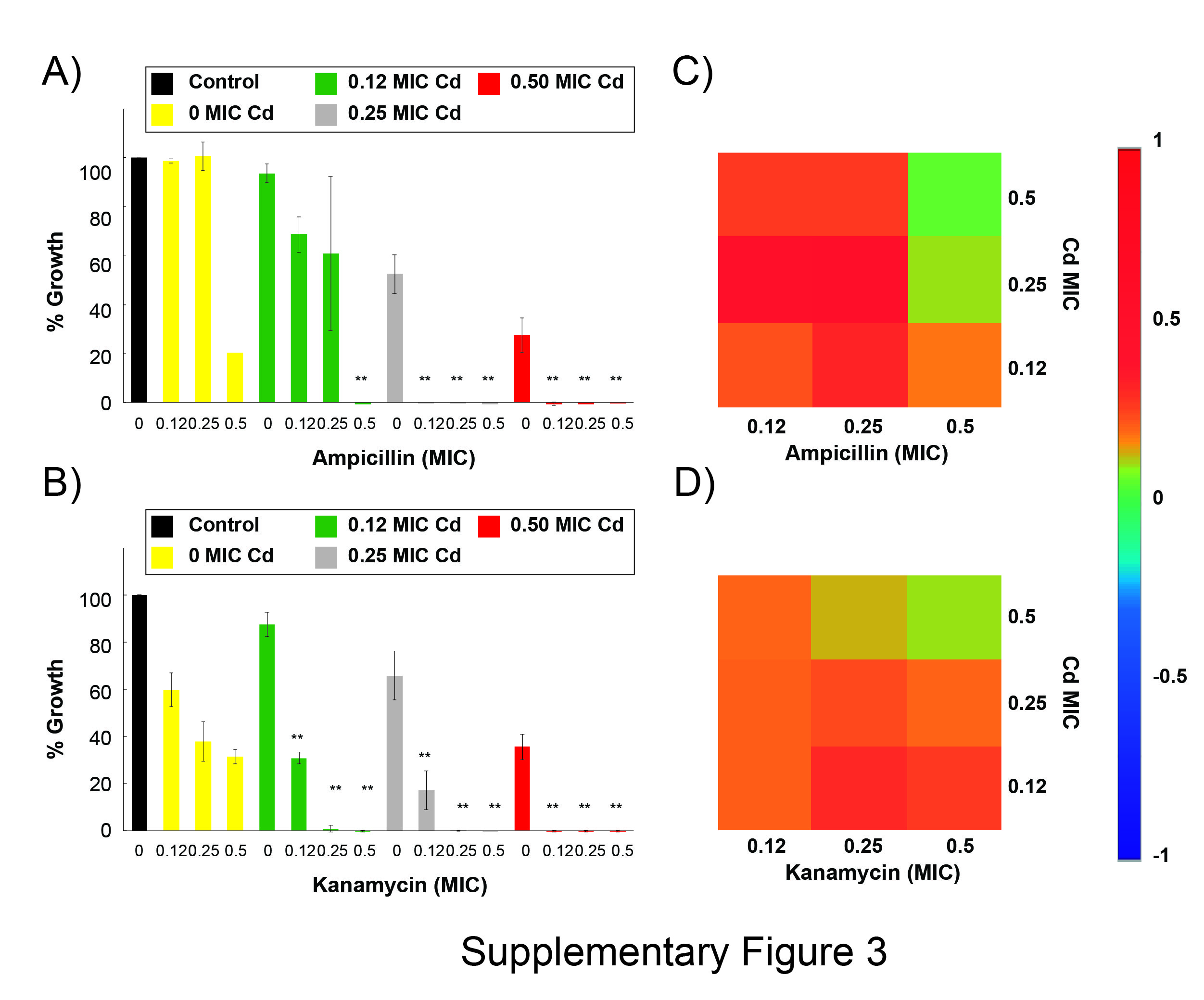


**SFigure 3.** Antimicrobial effect and interactions of ATMCs in antibiotic resistant *E. coli*. Growth percentage by sub-inhibitory concentrations of metal-antibiotic combinations: (**A**) Cd^2+^-ampicillin and (**B**) Cd^2+^-kanamycin against *E. coli-Amp* and *E. coli-Kan*, respectively. Classification of the different interactions between metal-antibiotic combinations. The interactions of (**C**) Cd^2+^-ampicillin and (**D**) Cd^2+^-kanamycin combinations are classified as synergistic, additive or antagonist, value >0, =0 and <0, respectively. Each experiment was done in triplicates. **Corresponds to a significant difference (p<0.05) with respect to the control and each of the individual treatments. Error bars corresponds to the standard deviation from experiments performed in triplicates.


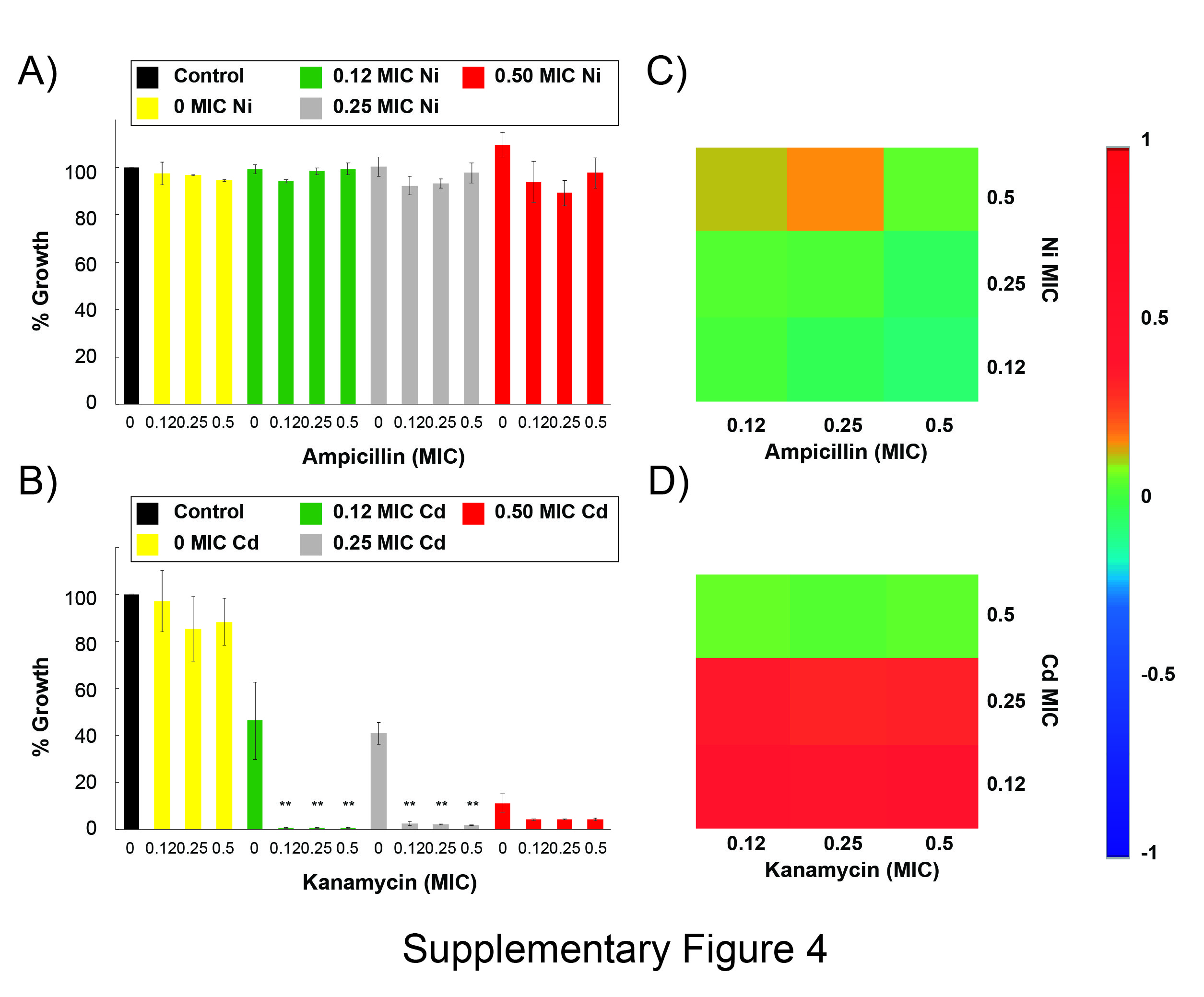


**SFigure 4.** Antimicrobial effect and interactions of ATMCs in antibiotic resistant *S. aureus*. Growth percentage by sub-inhibitory concentrations of metal-antibiotic combinations: (**A**) Ni^2+^-ampicillin and (**B**) Cd^2+^-kanamycin against *S. aureus-Amp* and *S. aureus-Kan,* respectively. Classification of the different interactions between metal-antibiotic combinations. The interactions of (**C**) Ni^2+^-ampicillin and (**D**) Cd^2+^-kanamycin combinations are classified as synergistic, additive or antagonist, value >0, =0 and <0, respectively. Each experiment was done in triplicates. **Corresponds to a significant difference (p<0.05) with respect to the control and each of the individual treatments. Error bars corresponds to the standard deviation from experiments performed in triplicates.
